# Improving cereal grain carbohydrates for diet and health

**DOI:** 10.1016/j.jcs.2014.01.001

**Published:** 2014-05

**Authors:** Domenico Lafiandra, Gabriele Riccardi, Peter R. Shewry

**Affiliations:** a Università degli Studi della Tuscia, Department of Agriculture, Forestry, Nature and Energy, Via S.C. De Lellis, Viterbo 01100, Italy; b Università degli Studi di Napoli Federico II, Department of Clinical Medicine and Surgery, Via Pansini 5, Napoli 80131, Italy; c Department of Plant Biology and Crop Science, Rothamsted Research, Harpenden, Hertfordshire AL5 2JQ, UK; d School of Agriculture, Policy and Development, University of Reading, Earley Gate, Whiteknights Road, Reading RG6 6AR, UK

**Keywords:** Cereals, Starch, Arabinoxylan, Beta-glucan, Dietary fibre, Health benefits

## Abstract

Starch and cell wall polysaccharides (dietary fibre) of cereal grains contribute to the health benefits associated with the consumption of whole grain cereal products, including reduced risk of obesity, type 2 diabetes, cardiovascular disease and colorectal cancer. The physiological bases for these effects are reviewed in relation to the structures and physical properties of the polysaccharides and their behaviour (including digestion and fermentation) in the gastro-intestinal tract. Strategies for modifying the content and composition of grain polysaccharides to increase their health benefits are discussed, including exploiting natural variation and using mutagenesis and transgenesis to generate further variation. These studies will facilitate the development of new types of cereals and cereal products to face the major health challenges of the 21st century.

## Highlights

• Resistant starch and cell wall polysaccharides have important impacts on human health.
• These effects are reviewed and their mechanisms discussed.
• Improved crops can be developed by classical breeding, mutagenesis and transgenesis.

## 1 Introduction

Cereals are the dominant crops in world agriculture, with a total of 2500 million tonnes being harvested globally in 2011, comprising 704 million tonnes of wheat, 723 million tonnes of rice, 883 million tonnes of maize (http://faostat.fao.org/site/291/default.aspx.) and lesser amounts of a number of “minor cereals” (barley, oats, rye, sorghum and millets). They are the major source of calories and protein to the diets of humans and livestock (including poultry).

Major reasons for the success of cereals include their adaptability, high yields, and ease of harvest and storage, but their processing and eating properties are also important, with wheat in particular being processed into a range of foods including bread and other baked goods, noodles and pasta. Failure of cereal harvests due to adverse weather, pathogens or human actions has contributed to famines in many countries, and increasing cereal production is a key aim of national and international research efforts.

Although emphasis is rightly placed on increasing crop production to meet the needs of the growing global population it is important to also bear in mind that the major effect of food on the health of the population in the developed world, and increasingly in rapidly developing economies such as India and China, relates not to lack of food but to over-consumption, particularly increased consumption of highly refined foods when associated with an increasingly sedentary life style. These include many starch-rich foods derived from cereals, which has conveyed a negative view of the contribution of cereals to diet and health to many consumers. In fact, this is far from the truth with cereals being important sources of many essential or beneficial components to the human diet. For example, the National Diet and Nutrition Survey of the UK showed that cereal products contributed 29/30% of the total daily energy intake of adult males/females, 22/21% of the intake of protein and 39/37% of the intake of non-starch polysaccharides (the major components of dietary fibre, DF) (NDNS, 2011). Similarly, Steer et al. (2008) showed that bread products alone contributed 12% of the protein, 20% of the fibre and 16% of the iron to the adult UK diet.

The present article therefore focuses on the contributions of cereal carbohydrates to a healthy diet, focussing on wheat with comparative data on other cereals being included where appropriate.

### 1.1 Introduction to grain carbohydrates

Carbohydrates can be classified according to their molecular size and degree of polymerisation, with each group being subdivided according to the number and composition of monosaccharide units. This classification includes sugars (monosaccharides and disaccharides), oligosaccharides, starch (amylose and amylopectin) and non-starch polysaccharides.

Carbohydrates account for about 65–75% of the mature wheat grain (Stone and Morell, 2009), with similar values being reported for other major cereals. Values reported for the amounts of individual groups in the wheat grain vary between different studies but are in the order of about 1% or less monosaccharides (glucose, fructose) and disaccharides (sucrose and maltose), about 1% oligosaccharides (raffinose and fructo-oligosaccharides), 1–2% fructans, 65–75% starch and about 10% cell wall polysaccharides (mainly cellulose, arabinoxylan and β-glucan), the latter forming the major DF components (Stone and Morell, 2009; Andersson et al., 2013). However, there are large differences between the compositions of the different grain tissues. In particular, the aleurone and outer layer (pericarp and testa), which form the bran fraction on milling of wheat, contain little starch but up to half of the dry weight is cell wall polysaccharides, while the starchy endosperm (the major storage tissue of the grain) comprises about 85% starch and only 2–3% cell wall polysaccharides.

Starch is composed of two different types of glucan polymers: amylopectin and amylose, present in a 3:1 ratio. Both polymers are formed of α-d-glucose but they differ in their level of branching, with amylose being essentially linear and amylopectin being highly branched. These branches can directly affect starch characteristics and functionality as differences in their chain length distribution and clustering, and the ability to form a double helical conformation, may contribute to the crystalline features of the amylopectin (Jeon et al., 2010).

**Fig. 1:**
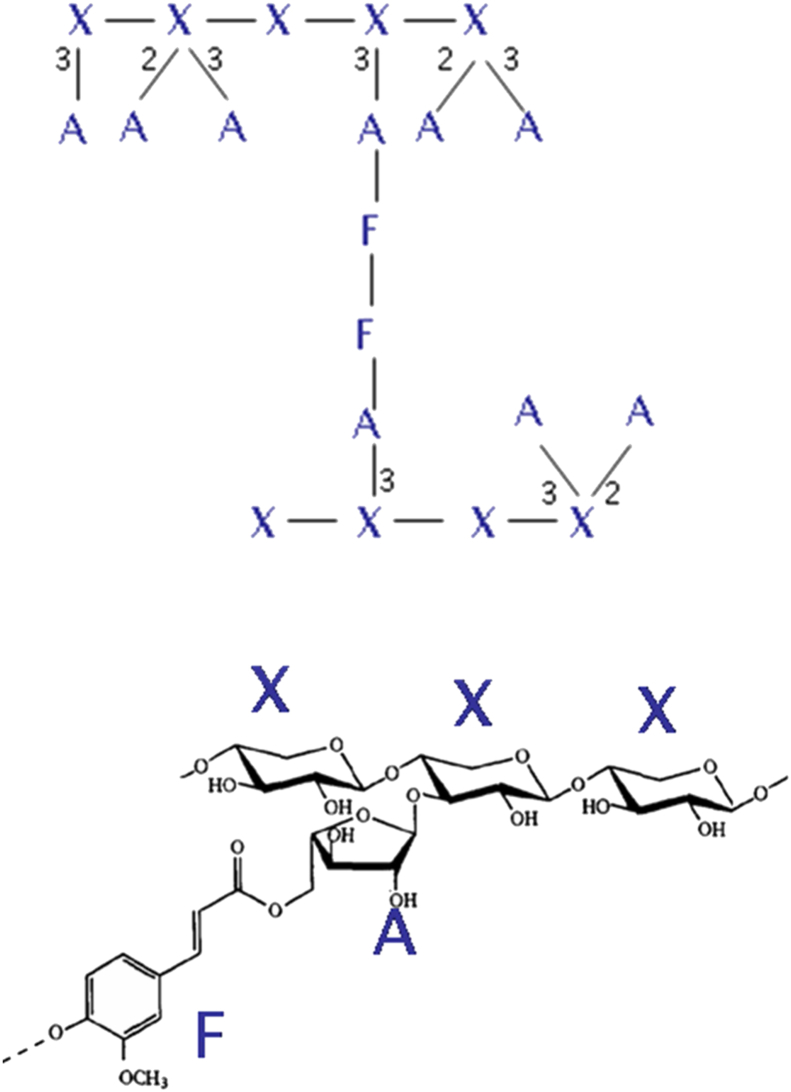
Schematic (top) and detailed (bottom) structures of wheat starchy endosperm arabinoxylan.

The major cell wall polysaccharides in the cell walls of wheat and related cereals (barley, oats and rye) grain are arabinoxylan (AX) and (1 → 3, 1 → 4)-β-d-glucan (β-glucan). AX comprises a backbone of β-d-xylopyranosyl residues linked through (1 → 4) glycosidic linkages, with some residues being substituted with α-l-arabinopyranosyl residues at either position 3 or positions 2 and 3 (Fig. 1). Substitution at position 2 alone occurs only rarely in wheat AX (Stone and Morell, 2009). The arabinose residues at position 3 of monosubstituted xylose residues may also be substituted with ferulic acid at the 5 position, allowing the formation of cross-links by oxidation of ferulate present on adjacent AX chains. This results in the formation of dehydrodiferulate (ferulate dimers) or, more rarely, triferulate residues which are stable to acid hydrolysis. A range of other modifications may also occur, including acetylation, substitution with galactose and glucuronic acid and substitution of the arabinose with *p*-coumaric acid instead of ferulic acid.

By contrast, β-glucan has a simpler structure, comprising only glucose residues which are linked by (1 → 4) and (1 → 3) bonds. In general, single (1 → 3) linkages are separated by 2 or 3 (1 → 4) linkages but the linkage ratio may vary between tissues and species and longer “cellulose-like” regions of continuous (1 → 4) linkages (up to 14) have been reported in wheat bran β-glucan (Li et al., 2006). The ratio between (1 → 4) and (1 → 3) linkages differs between different cereal species (Cui et al., 2000; Lazaridou et al., 2004; Lazaridou and Biliaderis, 2007), with the relative proportion of trisaccharide (DP3) decreasing from wheat (67–72%), to barley (52–69%) and oats (53–61%). It is probable that such variation in linkage pattern determines the differences that occur in the properties, including the solubility and viscosity, of β-glucans from various sources.

## 2 Contributions of grain carbohydrates to diet and health

### 2.1 Nutritional value of grain carbohydrates

All organisms require energy to maintain their life cycles. For humans, carbohydrates are the major energy sources, typically accounting for 45–70% of the total energy intake and expenditure. Being quantitatively the most important dietary energy source for most populations, carbohydrates have a special role to play in energy metabolism and homeostasis. Cereals are the dominant source of carbohydrates in the human diet, providing the major source of energy and contributing significantly to protein intake.

In addition to their classification based on degree of polymerisation and sugar composition, carbohydrates can also be classified based on the extent to which they are digested and absorbed in the human small intestine, thus contributing directly or indirectly to the body carbohydrate pool and influencing post-prandial glycaemia (glycaemic carbohydrates). In this classification, carbohydrates which are not digested and absorbed in the human small intestine are distinguished from glycaemic carbohydrates, forming the major part of DF (Mann et al., 2007).

The carbohydrates that have the greatest impact on post-prandial blood glucose are those that are absorbed relatively quickly from the small intestine. In fact, most metabolic parameters are adversely affected by an abrupt perturbation in the post-prandial period of a metabolic steady state present in the fasting condition. The greater and more rapid the perturbation, the more pronounced are the effects. Therefore this metabolic perturbation could be minimised if carbohydrate digestion and absorption were slowed down. This has resulted in interest in understanding the properties of foods that are able to retard digestion. The type of starch is certainly relevant in this respect, with amylose being slowly digested by α-amylases present in the human duodenum and amylopectin being very rapidly digested because the branched structure provides multiple sites for enzymatic hydrolysis. Moreover, amylose is structurally organised in the form of the double helixes, with the inner part of the helix accommodating the hydrophobic ends of polar lipids which form molecular complexes and reduce the accessibility of the molecule to α-amylase (Birt et al., 2013). These properties of amylose, in addition to its propensity to retrogradation, justify interest in producing high amylose wheat varieties for the preparation of cereal foods with a low glycaemic response (Asp et al., 1996; Saris et al., 1998; Rahman et al., 2007).

The contents of fibre and other food constituents that affect carbohydrate digestibility also affect the rise in blood glucose that follows the ingestion of carbohydrates from different foodstuffs, resulting in wide variation between different foods. The glycaemic response to food is also influenced by the method of preparation and other components of the meal.

The structure of food has long been recognised as an important parameter governing the health benefits of cereal products, in that the intact structure of a plant food is more important than the composition (including the amount of DF) in determining physiological responses such as those related to glucose metabolism. Many studies have shown the importance of preserving as much as possible of the natural network of cell wall polysaccharides present in plant tissues, particularly in more or less intact cereal (wheat, barley, rye and oat) grains (Riccardi et al., 2008).

A simple marker for the impact of carbohydrate-rich foods on post-prandial glycaemia is the glycaemic index. This index is based on the increase in blood glucose concentrations (the incremental area under the curve of blood glucose concentrations) after the ingestion of a portion of a test food containing 50 g of carbohydrate, divided by the incremental blood glucose area achieved with the same amount (50 g) of carbohydrate present in an equivalent portion of reference food (glucose or white bread). Fibre-rich foods often have a low glycaemic index, although some foods with a low fibre content may also have a low glycaemic index.

Low glycaemic index/high fibre diets have been shown to have beneficial effects, including lower post-prandial glucose and insulin responses, improved lipid control, and possibly improved insulin sensitivity. Although it is not possible to define precise values for high or low glycaemic index diets, it is clear that the lower the glycaemic index, the greater the metabolic effects (Saris et al., 1998).

### 2.2 Dietary fibre and health effects of cereal foods

Dietary fibre is one of the major food constituents that influence the rate and extent to which blood glucose increases after ingestion of a carbohydrate food. DF is not digested and absorbed in the small intestine but passes to the colon where it undergoes bacterial degradation. The principal types of DF are the non-starch polysaccharides (NSP), resistant starches (RS), non-α-glucan oligosaccharides (short chain carbohydrates) and some polyols and modified starches. The daily intake of NSP in the diet in European countries varies between 11 and 33 g, all of which will reach the colon (Saris et al., 1998).

An important health benefit of DF is the capacity to lower the glycaemic response of the foods in which it is present: foods rich in DF release glucose more slowly into the blood which is relevant to the prevention of disorders such as obesity and diabetes (Nugent, 2005).

Food viscosity is a potential mechanism by which DF slows down the rates of digestion of starch and the absorption of sugars. Soluble, viscous types of NSP present either in food raw materials or incorporated as ingredients are able to reduce the post-prandial glucose and insulin responses after a meal. The effect is considered to be related to viscosity, which inhibits mixing and diffusion in the intestinal tract and delays gastric emptying. Moreover, high viscosity decreases enzyme diffusion and stimulates the formation of an unstirred water layer which decreases glucose transport to enterocytes. It has been repeatedly demonstrated that reducing the viscosity of DF following acid hydrolysis results in concurrent loss of its clinical efficacy (Riccardi et al., 2004). Several types of soluble fibre, including β-glucan, psyllium and guar gum, reduce postprandial glucose and insulin responses, and improve insulin sensitivity both in diabetic and non-diabetic individuals (De Natale et al., 2012; Riccardi et al., 2005).

β-glucan varies in solubility, with a higher proportion of the total fraction being soluble in oats and barley than in wheat. The basis for this variation is still not completely understood, but it is likely that chain length (shorter chains being more soluble) and the ratio and distribution of 1 → 3 and 1 → 4 linkages both contribute. A significant relationship between the amount of β-glucan in cereals and the plasma glucose peak, or area under the glucose curve, has been observed. Similarly, a linear dose-dependent decrease in glycaemic responses was noted in response to breads containing proportions of barley β-glucan ranging from 0.1% to 6.3%. It has been suggested that viscosity of β-glucan could account for 79–96% of the effects on glucose and insulin responses after consumption of 50 g glucose in a drink model (El Khoury et al., 2012; Fardet, 2010).

Reduced insulin responses have consistently been observed following the ingestion of β-glucan and other soluble viscous types of fibre; as in the case of glycaemia, dose is an important factor in determining insulin responses to β-glucan. A consistent dose-dependent decrease in insulin secretions was observed in overweight individuals in response to oat β-glucan, with significant changes reported at a dose of at least 3.8 g of β-glucan (El Khoury et al., 2012). DF present in cereals also affects the intestinal absorption of other macronutrients, such as fat, although there is little evidence for significant effects in humans. Cereal β-glucan exhibits similar hypocholesterolemic effects on plasma lipids to those of other types of soluble dietary fibre (Poli et al., 2008).

A significant proportion of DF that enters the large bowel will be fermented by the commensal bacteria that live in the colon. Fermentation is an anaerobic process and, therefore, produces the short chain fatty acids (SCFA) acetate, propionate and butyrate as the principal end-products, together with methane, hydrogen, carbon dioxide and lactate. SCFA can influence liver glucose production and, thus, the plasma glucose concentrations (Slavin, 2013).

Among SCFA, butyrate has attracted particular interest because it is associated with unique benefits, such as the ability to promote a normal phenotype in colonocytes by repairing damaged DNA (Le Leu et al., 2005; Toden et al., 2007; Topping et al., 2008) and inducing death in transformed cells (Candido et al., 1978). These mechanisms are believed to be effective in the prevention of serious diseases such as colorectal cancer.

Butyrate is the major energy source for the colonic epithelial cells and also has differentiating properties in the cells, arresting cell division through its ability to regulate gene expression. This property provides a credible link between the dietary intake of fermented carbohydrates, such as NSP, and protection against colorectal cancer (Roberfroid et al., 2010).

Propionate is absorbed and passes to the liver where it is taken up and metabolised aerobically. This molecule moderates hepatic lipid metabolism. Acetate is the major SCFA produced in all types of fermentation, the molar ratio of acetate:propionate:butyrate usually being around 60:20:20. Acetate is rapidly absorbed, stimulating sodium absorption, and passes to the liver and then into the blood from where it is available as an energy source. The fasting levels of blood acetate are about 50 μmol/L, rising to 100–300 μmol/L between 8 and 12 h after meals containing fermentable carbohydrate. Acetate is rapidly cleared from the blood with a half-life of only a few minutes and is metabolised principally by the skeletal and cardiac muscle and the brain. Acetate reduces free fatty acid oxidation in humans and its absorption does not stimulate insulin release (Liljeberg and Björck, 1996; Saris et al., 1998).

Foods containing high levels of fibre are often made from whole grains or whole foods such as fruit and vegetables and are thus bulky with a low energy density. This may promote satiety and therefore may be expected to encourage weight loss, or prevent weight gain. In addition, some types of fibre, present in oats, barley, pulses, vegetables and fruit, are viscous and may act to delay gastric emptying and reduce the rate of absorption of glucose and fat from the small intestine, which may further contribute to feelings of increased fullness and satiety. The colonic metabolism of fibre after ingestion of the meal may delay the onset of hunger before the next meal as a consequence of the production of short-chain fatty acids and other satiety signals (Riccardi et al., 2004).

A further mechanism by which whole grain may influence body weight regulation is via a probiotic effect modulating the intestinal flora. The available evidence, primarily from studies in animal models, suggests that the gut microbiota affect nutrient acquisition and energy regulation. Microbiota composition has also been shown to differ in lean vs obese animals and humans. Of particular interest among the possible mechanisms determining this relationship is the hypothesis that the metabolic activities of the gut microbiota facilitate the extraction of calories from ingested dietary substances and help store these calories in the host adipose tissue for later use. In fact, the gut bacterial floras of obese mice and humans include fewer Bacteroidetes and correspondingly more Firmicutes than those of their lean counterparts, suggesting that differences in the extraction of calories from ingested food substances may be due to the composition of the gut microbiota. The focus on Bacteroidetes, however, seems to be controversial. It has been hypothesised that more specific modulations of the gut microbiota community occur in obesity (instead of those at the wide phylum levels); this hypothesis is supported by several studies (Delzenne et al., 2011). It is not known whether the habitual intake of whole grain products is able to influence the composition of the gut microbiota; however, available data in humans indicate that a diet rich in whole wheat cereals is associated with an increased number of faecal Bifidobacteria and Lactobacilli, the target genera for prebiotic intake (Delzenne et al., 2013; Giacco et al., 2011).

### 2.3 Grain carbohydrates and prevention of metabolic and cardiovascular diseases

Epidemiological observations consistently show an association between consumption of whole grain cereal foods and prevention of several non-communicable diseases; in particular, several studies show that subjects who ingest three or more portions of foods per day based on whole grain cereals have a 20–30% lower risk of CVD than subjects who consume predominantly refined cereals. Similarly, a high intake of whole grain cereals and their products, such as whole wheat bread, is associated with a 20–30% reduction in the risk of type 2 diabetes (Gil et al., 2011).

Several prospective studies have also shown a more general inverse relationship between the consumption of fibre-rich foods and the risk of type 2 diabetes. It is noteworthy that in all of these studies the intake of cereal fibre in particular was inversely associated with the incidence of diabetes (Parillo and Riccardi, 2004). In fact, the regular consumption of whole grain cereal foods has been found to be consistently associated with a lower risk of developing overweight, type 2 diabetes and cardiovascular diseases in both cross-sectional and longitudinal epidemiological studies (Riccardi and Rivellese, 2000).

No intervention studies have so far determined the effects of fibre, glycaemic index and/or whole grains on the prevention of diabetes, although the most important independent studies aimed at lifestyle modification also included increases in fibre consumption and, in particular, consumption of whole grain fibre in the intervention group.

Results from human intervention studies with surrogate end points consuming whole grain cereal products are not always consistent since the outcomes may be influenced by a number of factors such as the type of cereals consumed and their fibre contents together with other dietary factors which may be relevant for health such as the polyphenol content and the overall diet composition (Cho et al., 2013).

The benefits of regular intake of whole grain and cereal fibre may be mediated by the improvement of one or more risk factors for type 2 diabetes and cardiovascular diseases, such as insulin resistance, dyslipidemia, inflammation and oxidative stress (Wirstrom et al., 2013). However, intervention studies of the effects of whole grain on the regulation of glucose/insulin metabolism have thus far provided conflicting results. Some clinical trials have shown an improvement in insulin sensitivity, while other studies have reported no effect on either glucose or insulin metabolism (Brownlee et al., 2010). Similarly, conflicting data are available on the effects of increased whole grain consumption on C-reactive protein (CRP) and other markers of inflammation (Giacco et al., 2010). As for the effects of whole grain consumption on lipid metabolism, there is a general consensus that whole grain cereals rich in β-glucan, such as oats and barley, have effects on the reduction of fasting plasma concentrations of both total and LDL cholesterol. However, clinical trials with whole wheat and/or whole grain rye products have reported mixed results (Giacco et al., 2013; Juvonen et al., 2011; Rosén et al., 2011).

It is possible that the benefits of whole grain consumption in reducing the risks of type 2 diabetes and CVD could also be mediated by mechanisms that have not yet been investigated, particularly in relation to the post-prandial metabolism. A large body of evidence indicates that metabolic abnormalities in pre-diabetic insulin-resistant subjects and in diabetic patients are related more tightly to the post-prandial condition than to the fasting state. Indeed, increases in blood glucose, insulin and lipids in the post-prandial period are risk factors for adverse cardiovascular events that can also be detected in the absence of altered fasting parameters (Saris et al., 1998). It can be hypothesised that whole grain intake exerts its metabolic effects mainly during the post-prandial period with minimal impact, at least in the short/medium term, on fasting parameters. In this regard, very few studies have focused on investigating the effects of whole grain cereals on post-prandial metabolism. In fact, in acute experimental settings, a reduction in insulin response has been reported with whole kernel rye/whole rye bread when compared with white wheat bread. This has been confirmed in longer term experimental conditions (2–4 weeks) that demonstrated a reduction of both insulin and glucose post-prandial responses after a whole grain rye or wheat diet in overweight men (Gil et al., 2011).

### 2.4 Resistant starch (RS)

The digestibility of starch is of great importance to human health (Copeland et al., 2009) with a variable proportion remaining undigested in the small intestine and reaching the large intestine as part of the DF fraction where it is fermented by the gut bacterial microflora. This “resistant starch” (RS) has been defined as “the starch and products of starch digestion that are not absorbed in the small intestine of healthy individuals” (Englyst and Cummings, 1985; Asp, 1992) and is subdivided into five categories: physically inaccessible starch (RSI), resistant starch granules (RSII), retrograded starch (RSIII), chemically modified starch (RSIV) and starch capable of forming complexes between amylose and long branch chains of amylopectin with lipids (RSV) (Thompson, 2000; Sharma et al., 2008; Hasjim et al., 2010; Birt et al., 2013). In fact, the resistance of starch to digestion is influenced by many properties of the starch granule, such as: size, shape and crystallinity, lipid, protein and phosphate contents and amylose content (Themeier et al., 2005; Tester et al., 2006). In addition, food processing may affect starch digestibility (Alsaffar, 2011). In terms of crop modification, attention has been focused almost entirely on changing the proportions of low branched (amylose) and highly branched (amylopectin) polymers.

RS has beneficial physiological effects (Nugent, 2005) associated with metabolic products released during its fermentation in the large bowel, particularly the production of the SCFAs butyrate, acetate and propionate. These are important metabolites for viscera contributing to their energetic needs (Topping, 2007). The beneficial effects of the fermentation of RS2 from high amylose maize have recently been demonstrated by a microarray study carried out with a total RNA extract from caecal cells in rats (Keenan et al., 2012). Fermentation of RS resulted in altered expression of genes involved in cell growth, proliferation and differentiation of the gut, as well as the expression of genes involved in apoptosis and the control of cell proliferation. The increased gene expression also appeared to improve the structure and function of the gastro-intestinal (GI) tract. SCFAs also play an important role in the lowering of the pH in the GI tract, which contributes to control of the proliferation of pathogenic bacteria and avoids the formation of harmful cytotoxic compounds.

An improvement of blood glucose regulation has been associated with the consumption of cereals rich in RS (Akerberg et al., 1998; Granfeldt et al., 1995; Li et al., 2011; Hallström et al., 2011). RS is similar to NSP in that it can delay gastric emptying, reduce the rate of glucose absorption and is fermented by colonic bacteria producing SCFA (Slavin and Green, 2007; Pereira and Ludwig, 2001; Bodinham et al., 2010).

The metabolism of RS in the large bowel is beneficial because it is associated with the production of higher levels of butyrate compared to other types of dietary fibre (Topping and Clifton, 2001; Topping et al., 2008). RS can also help to control body weight since it is able to prolong satiety, due to its low energy density, and its ability to be fermented by colonic bacteria producing SCFAs that are able to reduce the energy intake (Slavin and Green, 2007; Pereira and Ludwig, 2001; Bodinham et al., 2010). According to Keenan et al. (2006), the consumption of resistant starch in the diet represents also a natural, endogenous way to increase gut hormones, such as glucagon-like peptide (GLP-1) and peptide YY (PYY) that are effective in reducing energy intake. The PYY and GLP-1 signals associated with use of RS in the diet may alter the long-term energy balance by interacting with neuronal pathways in the brain and also have other benefits such as improved gut health (Slavin, 2013).

In addition to its important impact on health, RS also confers improved quality features to several food products. RS has the desirable properties of low water binding capacity, small particle size, and bland flavour, and has a positive impact on dough rheological characteristics and product palatability (Sharma et al., 2008). Aravind et al. (2013) compared the technological, sensory, and structural properties and *in vitro* starch digestibility of pasta enriched with commercial resistant starches at 10%, 20% and 50% for RS2 and 10% and 20% for RS3 with pasta made from 100% durum wheat semolina, The resultant RS content of pasta was increased from 1.9% to 21% in the blend made with 50% RS2 and was not reduced on cooking. Significantly, the results indicated that substitution of semolina with 10% and 20% RS2 and RS3 had no significant effects on pasta loss on cooking, texture and sensory properties, with only a minimal reduction in pasta yellowness. The incorporation of commercial high amylose starch in baked products such as cakes, muffins and biscuits has resulted in significant increases in total DF content and, at the same time, softer texture (Waring, 2005). RS has also been shown to enhance pasta firmness and to increase expansion, crispness and oil absorption of snack foods (Sajilata and Singhal, 2005; Sajilata et al., 2006; Soh et al., 2006).

The demonstration that the amount of RS2 and the ability to form RS3 are influenced by the amylose content (Morell et al., 2004; Rahman et al., 2007) has resulted in interest in increasing resistant starch through the manipulation of genes involved in the starch biosynthetic pathway, using classical or novel breeding approaches.

## 3 Strategies to improve grain composition

In the past century, breeders have aimed to develop highly productive and uniform varieties of wheat and other crops, which has resulted in decreased genetic diversity. It is therefore necessary to seek ways to increase the range of diversity in key traits.

### 3.1 Exploiting natural variation

Classical plant breeding depends on exploiting natural variation, which is generated by recombination during crossing of genotypes with different genetic backgrounds. The range of variation which is available varies depending on the trait, and may also be modulated by interactions between the genotype and environment. Wider variation may also be available in species related to the crop of interest, and exploited using “wide crossing”. Depending on the degree of relationship and sexual compatibility of the crop and wild relative, this may require the use of embryo rescue or tissue culture to recover fertile embryos. An extensive programme of back crossing is also required to eliminate deleterious genes from the wild relative which are not related to the trait of interest.

### 3.2 Mutagenesis and TILLING

Mutation breeding, which is based on the use of radiation or chemical agents to induce mutations, has proved to be very effective in generating novel genetic diversity for important traits that has been exploited in breeding programmes. This has resulted in the production of 3218 different crop cultivars (http://mvgs.iaea.org/Default.aspx) with economic and social benefits. More recently, the availability of large genomic resources and bioinformatic tools together with new sophisticated technologies to detect mutations in target genes have led to the development of a technology termed TILLING (Targeting Induced Local Lesions in Genomes). This approach combines classical chemical mutagenesis and PCR-based screening to identify mutations in a given gene of interest (McCallum et al., 2000). TILLING involves the use of chemical agents such as sodium azide (NaN_3_) or ethyl methane sulfonate (EMS) which are able to alkylate nucleotides to induce point mutations which are randomly distributed over the entire genome. EMS alkylates guanine bases that will then pair with thymine instead of cytosine, ultimately resulting in a G/C to A/T transition. Different high-throughput technologies are available for mutation detection (Sikora et al., 2011). Treatment of seeds with EMS is very effective in producing nonsense, missense and silent mutations. Nonsense mutations can cause loss of gene function, with truncation or loss of expression of corresponding protein; whereas missense mutations result in the change of an amino acid in the protein encoded by the mutated gene. Missense mutations can also affect the functionality of the protein if they occur in the catalytic domain or affect the protein folding or assembly. Silent mutations have no effect on the protein sequence or functionality. Comai et al. (2004) also used TILLING to identify genetic variation in natural populations and termed this approach EcoTILLING.

Mutagenised populations and TILLING platforms have been developed for cereals and are being used in functional genomic studies and practical breeding. In addition, as noted by Sikora et al. (2011), mutagenised populations can be directly screened for variation in the content and composition of components including starch.

### 3.3 Transgenesis

Transformation systems are now available for the major cereals (wheat, rice and maize) and most minor cereals (including barley, oats, triticale, sorghum and tef) (Jones and Shewry, 2008; Dunwell and Wetten, 2012), with transgenic maize being grown on over 50 million ha in 17 countries in 2012 (http://www.isaaa.org/inbrief/default.asp). This includes 129,000 ha of transgenic insect resistant maize in five EU countries. Furthermore, transgenesis is increasingly being applied to improve grain composition and quality, as reviewed by Dunwell (2014) in this issue. Hence, the main barrier to the deployment of transgenesis to improve the health benefits of cereal grain is low acceptability by consumers and regulatory authorities, with no nutritionally enhanced cereals currently being grown for human consumption.

## 4 Improving the starch composition of cereals for health benefits

### 4.1 Variation in starch composition

Starch is deposited within amyloplasts in discrete semi-crystalline structures termed granules. Starch granule size and shape vary greatly among the different types of cereals. The starch granules present in members of the *Triticeae* tribe, wheat, barley and rye, have a bimodal size distribution with larger ellipsoidal A-type granules ranging from 10 to 40 μm in diameter and smaller spherical B-type granules between 2 and 10 μm. Starch granules in maize have a polyhedral or spherical structure, with a size ranging between 2 and 30 μm, whereas rice granules exhibit a polyhedral shape and size ranging between 3 and 8 μm (Tester et al., 2004, 2006).

The relative amounts of amylose and amylopectin and their molecular structures contribute to its unique properties with strong effects on specific end uses in the food and manufacturing industries (Blennow, 2004; Blazek and Copeland, 2008; Copeland et al., 2009). Data on the role of starch in the prevention of chronic diseases has stimulated interest in the manipulation of starch structure in order to increase the health benefits.

**Fig. 2:**
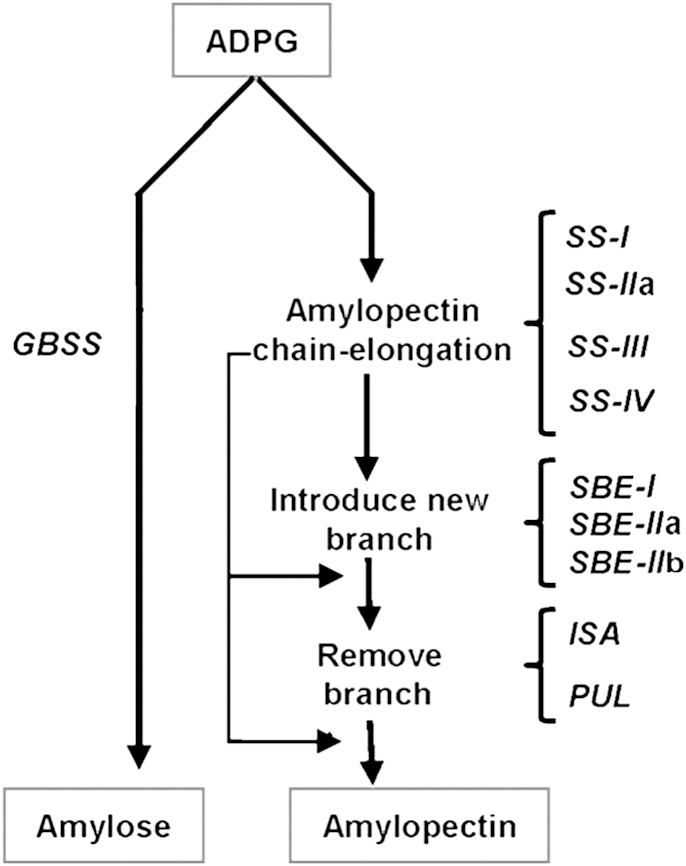
Pathway of starch biosynthesis in wheat. Modified from Tian et al. (2009) with permission.

Genetic modification of starch composition in cereals, through modulation of the amylopectin/amylose ratio, has become feasible thanks to the elucidation of its biosynthetic pathway. Genes encoding enzymes involved in the synthesis of the two different polymers have been identified using classical and biotechnological approaches. Starch synthases (SS), branching and debranching enzymes (SBE and DBE), which can be found in the soluble fraction of the endosperm, bound to starch granules, or both, are involved in amylose and amylopectin synthesis, through two different pathways which have ADP-glucose as a common substrate (Fig. 2). Detailed studies of the changes in structure and composition of starch in mutants lacking specific enzyme activities, have shed light on the contributions of different enzymes and led to the possibility of manipulation of genes involved in the starch biosynthetic pathway. This has made it possible to tailor starch composition in a predictable way for specific end uses (Blennow, 2004; Lafiandra et al., 2010; Blennow et al., 2013).

Detailed accounts of the role of the different enzymes involved in starch biosynthesis have been provided by several authors (Hannah and James, 2008; Stone and Morell, 2009; Jeon et al., 2010; Keeling and Myers, 2010; Zeeman et al., 2010; Tetlow, 2011) and only a short description will be given here.

A single starch synthase, the granule bound starch synthase (GBSS), is involved in the synthesis of amylose. This enzyme is also known as waxy protein reflecting the typical phenotypes of seeds lacking amylose. In diploid cereals such as maize, barley and rice, the inactivation of genes encoding GBSS results in starch containing only amylopectin. In polyploid species, such as bread wheat, the waxy phenotype may result from the complete inactivation of genes present at three different loci located on the short arms of chromosomes 7A and 7D and on the long arm of the chromosome 4A. The inactivation of one or two of these homoeologous genes results in the production of partial waxy lines (Graybosch, 1998) which can then be crossed to obtain partial and complete waxy genotypes in both bread and durum wheats (Graybosch, 1998; Lafiandra et al., 2010). Complete waxy lines are characterised by starch with a very low amylose content (0–2%) and unusual functionality, making them suitable for the production of refrigerated and frozen foods and as fat replacers. Miura et al. (1994) determined the differential effects of the three waxy protein isoforms on the amylose content and showed that the largest effect was associated with the Wx-B1 allele. Polymorphism at the waxy loci in durum and bread wheats, and its effect on the amount of amylose, has also been investigated by Yamamori (2009), identifying alleles associated with reduced and increased amylose content.

In addition to its role in amylose synthesis, GBSS has been suggested to play a role in the extension of the extra long chains of amylopectin (Yoo and Jane, 2002; Tetlow, 2011). Attempts have been made to relate waxy gene polymorphism with amylose content. The amylose content of starch in the endosperm is usually higher in *indica* rice than in *japonica* rice and this has been related to the presence of two types of *waxy* alleles, *Wxa* and *Wxb*. Rice varieties with the *Wxa* allele produce more GBSSI than varieties with the *Wxb* allele. The fact that many *indica* varieties possess the *Wxa* allele, while *japonica* varieties usually have the *Wxb* allele, therefore accounts for the higher amylose content in endosperm of *indica* rice than in *japonica* rice (Sano, 1984; Umemoto et al., 2002).

Amylopectin synthesis is more complex than the synthesis of amylose and involves several different enzymes. Four classes of SS (SSI, SSII, SSIII and SSIV) have been shown to contribute to amylopectin synthesis through effects on the synthesis of glucan chains of different length, with SSI appearing to play a direct role in the synthesis of short glucan chains (DP 6–12) (Tetlow, 2011).

Two different types of SSII genes are present in cereals, with one being expressed in the endosperm (SSIIa) and the other in the leaf (SSIIb). Li et al. (1999) have demonstrated that three starch granule proteins are present in bread wheat. These are called SGP-1(starch granule proteins-1), previously described by Yamamori and Endo (1996), and are encoded by the *SSIIa* genes present on the homeologous group 7 chromosomes. Loss of this activity in wheat, rice and barley (Yamamori et al., 2000; Umemoto et al., 2002; Morell et al., 2003; Konik-Rose et al., 2007) results in a characteristic phenotype comprising reduced starch content and swelling properties, increased amylose content, modified amylopectin chain length and reduced granule gelatinisation temperature. Genotypes lacking one of the three possible isoforms have been identified in wheat (Yamamori and Endo, 1996). Studies of SSIII enzymes have identified a possible role in the synthesis of amylopectin long chains and a possible regulatory role in starch synthesis. In addition mutations in this gene in maize, rice and barley have also been shown to result in an increased amylose level (Li et al., 2011). No clear role has been assigned to the SSIV enzymes (Tetlow, 2011). Branching of the glucan chains is catalysed by starch branching enzymes, through cleavage of the α-1,4 glucosidic linkage of a glucan chain and subsequent bonding of the cleaved portion of this glucan chain to an adjoining chain through an α-1,6 linkage (Keeling and Myers, 2010). Two major classes of SBE, SBEI and SBEII, have been identified in cereals (Stone and Morell, 2009; Tetlow, 2011). No major effects on starch composition and structure have been identified when SBEI activity is eliminated, although a regulatory role in influencing other SBEs has been suggested (Tetlow, 2011). Branching enzymes of class II include two genes designated *SBEIIa* and *SBEIIb*. In wheat, down-regulation of the *SBEIIa* gene results in a phenotype with high amylose content and modified granule morphology. There is a marked decrease in the proportion of chain lengths of DP 4–12 and a corresponding increase in the chain lengths greater than DP 12. Whereas no effect is associated with the loss of expression of *SBEIIb* in wheat (Regina et al., 2006; Sestili et al., 2010b), the absence of expression in maize and rice results in a phenotype, designated as amylose extender (*ae*) (Mizuno et al., 1993; Boyer and Preiss, 1978), with a profound effect on the amount of amylose and the structure of amylopectin.

It should be stressed that reduction or elimination of starch branching enzyme activity will not result in the production of true amylose; the major effect will be the reduction of branch points in the amylopectin fraction that will result in the formation of an amylopectin polymer with amylose-like properties (Morell et al., 2004).

In addition to the enzymes described above, two types of debranching enzyme (DBEs) have been identified in cereals: isoamylases and pullulanases (limit dextrinases). Both hydrolyse α-1,6 linkages of amylopectin but they differ in substrate specificity. The absence of isoamylase activity is associated with lower starch content, the formation of a highly branched polymer (phytoglycogen) and changes in the number and size distribution of starch granules (Kubo et al., 1999; Fujita et al., 2003; Stahl et al., 2004). In particular, a strong increase in the total number of granules has been observed, which are of irregular shape and intermediate in size compared with the A and B granules normally present in the *Triticeae*.

### 4.2 Natural and induced mutations affecting starch composition

In recent years the manipulation of the amylose-amylopectin ratio in cereals has been identified as a major target for the production of starches with novel functional properties and improved health benefits. Together with natural mutants, induced mutations have been extensively explored in order to extend the narrow range of genetic variation for starch characteristics in crops. The studies carried out on maize, rice, wheat and barley have indicated that lack of activity of starch synthase SSII or SBE (SBEIIa or SBEIIb) are more effective in increasing amylose content in the seeds.

Several mutants influencing the amount and composition of starch in maize have been identified since the middle of the twentieth century (Creech et al., 1963; Creech, 1965). The discovery of the amylose extender (*ae*) allele by Vineyard and Bear (1952), which increases the amylose content of starch without affecting significantly the total amount of starch in the grain, paved the way for intensive breeding, in both the private and public sectors, that resulted in the release of maize hybrids with desirable agronomic qualities and high amylose starch, termed “Amylomaize” (Fergason, 2001). Maize *ae* mutants lack SBEIIb activity (Hedman and Boyer, 1982; Liu et al., 2012). Subsequent studies have revealed that mutant *ae* alleles interact with an unknown number of genetic modifiers, contributing to the range of values of amylose found when different inbred lines are crossed (Fergason, 2001).

Commercial maize varieties possessing high-amylose starch have been classified into amylomaizes V, VI, and VII, reflecting amylose contents of 50, 60, and 70%, respectively (Vineyard et al., 1958; Jiang et al., 2010). More recently, breeding has resulted in the production of maize lines with amylose contents up to 85–90% (Campbell et al., 2007; Li et al., 2008).

In barley, the first high amylose mutant (45%) was identified by Merritt (1967) and the gene controlling the trait was designated *amo 1* by Eslick and Ullrich (1977). Treatment of seeds of the barley variety Himalaya 292 with sodium azide resulted in the identification of mutants with amylose content ranging from 65% to 70% (Morell et al., 2003). Lack of SSIIa protein was observed in the mutant lines and the DNA sequence of corresponding *SSIIa* gene showed the presence of a stop codon responsible for the mutation and resulting in inhibition of the translation of the transcript.

Chemical and physical mutagens have been used to induce mutations affecting starch biosynthesis in *japonica* rice varieties (Yang et al., 2006). Yano et al. (1985) used chemical mutagens to identify five mutant lines with amylose contents ranging from 29.4% to 32.4%, compared to 17.4% in the normal variety.

Treatment of the seed of the *japonica* rice variety Ilpumbyeo with *N*-methyl-*N*-nitrosourea resulted in the production of the new rice variety Goami 2 with an amylose content of 34%, compared to Ilpumbyeo that had 18.63% (Kang et al., 2003). According to Butardo et al. (2012), the higher amylose content of Goami 2 could be due to a mutation responsible for the trapping and accumulation of SBEIIb inside the starch granules in much greater amounts compared to Ilpumbyeo. This could prevent SBEIIb from carrying out its role in starch biosynthesis if its main function occurs at the surface of the starch granule.

SDS-PAGE of the starch granule proteins present in a large collection of bread wheat lines originating from different countries allowed Yamamori and Endo (1996) to identify mutants lacking each of the three possible homoeologous SSIIa proteins. The three different single null mutants were crossed to produce bread wheat lines lacking all the three starch synthases proteins by Yamamori et al. (2000). The starch contents of these lines were about 20% lower compared to that of normal wheat and the amylose contents 37.4%. Using the SGP-A1 and SGP-B1 mutants identified by Yamamori and Endo (1996), Lafiandra et al. (2010) produced partial and complete *null* lines in the Italian durum wheat cultivar Svevo. The complete SSIIa *null* line showed an amylose content of 43.6% compared to 23% in Svevo.

More recently, Hogg et al. (2013) analysed 255 *T. durum* accessions and identified two SGP-A1 nulls but no SGP-B1 nulls. The SGP-A1 nulls were crossed with the durum variety ‘Mountrail’ and F5-derived SGP-A1 null progeny lines were treated with EMS. One SGP-B1 null mutation was recovered from each EMS population, with each being a missense mutation. Each of the SGP-1 nulls was found to have large increases in amylose content (44.3% and 42.8% using an iodine colorimetric assay) compared to the wild type (28.7%).

As mentioned above, the development of TILLING technology has allowed the generation and detection of novel allelic diversity in genes involved in starch biosynthesis by several research groups. Slade et al. (2005) analysed two EMS mutagenised populations of durum and bread wheat, targeting the waxy genes and were able to detect 50 and 196 new alleles, respectively in durum and bread wheats.

TILLING resulted in the identification of more genetic diversity than had been reported by other research groups analysing wild and cultivated wheats. An EMS-mutagenised population of the bread wheat cultivar Cadenza was screened by Sestili et al. (2010a), combining SDS–PAGE analysis of starch granule proteins with TILLING. In particular, they focused on the *SSIIa* and *waxy* genes and found single *null* genotypes for each of the three homoeologous loci of the target genes. Another application of TILLING to modify starch composition in wheat has been reported by Uauy et al. (2009). These authors targeted genes encoding *SBEIIa* and *SBEIIb* in durum and bread wheat. Screening of the bread wheat TILLING population identified 65 mutations in *SBEIIa* alleles. In durum wheat, 58 and 35 mutants were detected for the *SBEIIa* and *SBEIIb* genes, respectively. The same genes have been targeted by TILLING in the bread wheat cultivar Cadenza by Botticella et al. (2011), who reported the identification of 123 novel allelic variants for the three homoeologous genes coding *SBEIIa*. The crossing of knock-out mutants gave a set of single and double *null SBEIIa* lines. Pyramiding of two *null* homeologs resulted in an increase in the amylose content by up to 21% compared to the control line Cadenza. Although total starch content decreased slightly, 100 seed weight did not differ significantly among the single and double *null* genotypes with respect to the control. More recently, Hazard et al. (2012) used a tetraploid wheat TILLING population, obtained by EMS treatment of the durum wheat cultivar Kronos, to identify mutations in the two *SBEIIa* and *SBEIIb* genes. Single gene mutants showed no significant increases in the contents of amylose and resistant starch, but when a double mutant was obtained, by combining a *SBEIIa*-A knock-out mutation with a *SBEIIa*-*B* splice-site mutation, an increase of 22% in amylose content and 115% in resistant starch content was observed.

Similarly, Slade et al. (2012) used TILLING to identify novel genetic variation in each of the A and B genomes of tetraploid durum wheat and the A, B and D genomes of hexaploid bread wheat, identifying deleterious mutations in the form of single nucleotide polymorphisms (SNPs) in *SBEIIa* genes. Combining these new alleles of *SBEIIa* resulted in high amylose durum and bread wheat lines containing 47–55% amylose and with elevated levels of resistant starch compared with wild type wheat. The high amylose lines also had reduced expression of *SBEIIa*, changes in starch granule morphology and altered starch granule protein profiles as evaluated by mass spectrometry.

Kadaru et al. (2006) described the application of EcoTILLING to detect allelic variation in *SSIIa* and *waxy* genes in 57 inbred rice accessions.

### 4.3 Using transgenesis to modify starch composition

RNA interference (RNAi), offers a robust approach to manipulate gene expression with the ability to simultaneously suppress members of multigene families, in order to modify crop quality traits (Jones et al., 2009) including starch composition. Regina et al. (2006) used RNAi technology to silence the *SBEIIa* genes and increase the amylose content of bread wheat. Suppression of *SBEIIa* resulted in high amylose contents of the transgenic lines (a value of 88.5% was obtained with iodometric determination and 77.4% using the size exclusion chromatography). The transgenic line showed altered starch granule morphology, a marked decrease in the proportion of chain lengths of DP 4–12 and a corresponding increase in the chain lengths greater than DP 12.

The same approach was used by Sestili et al. (2010b) to increase the amylose content of two durum wheat cultivars (Svevo and Ofanto). Although two different methods were used for genetic transformation (biolistic for cv. Svevo and *Agrobacterium* for cv. Ofanto), the silencing of *SBEIIa* genes produced similar effects. The *Agrobacterium*- and biolistic-mediated transformants showed various levels of silencing between independent transgenic lines. The abundance of mRNAs of *SBEIIa* genes varied in the transgenic lines from nearly undetectable to wild type level, confirming that the RNAi construct, designed to reduce the expression of *SBEIIa* genes, was effective in inducing gene silencing. An increase in amylose content was found in all transgenic lines but the percentage varied between lines, presumably due to differences in copy number and/or positional effects. One of the lines showed a high value of 75% amylose content, while in the other lines, the content varied between 31 and 56%.

In order to define the roles of different isoforms of SBEIIa and SBEIIb in determining amylose content, chain length distribution, and branching frequency of starch in barley, Regina et al. (2010) generated lines with low expression of SBEIIa or SBEIIb, and with the low expression of both isoforms through RNA-mediated silencing technology. These lines enabled the study of the role of each of these isoforms in determining the amylose content, the distribution of chain lengths, and the frequency of branching in both amylose and amylopectin. In lines where both SBE IIa and SBE IIb expression were reduced by >80%, a high amylose phenotype (>70%) was observed, while a reduced expression of either of these isoforms alone had little impact on amylose content. The structure and properties of the high amylose starch resulting from the concomitant reduction in the expression of both isoforms of SBEII in barley were similar to the changes in *ae* mutants of maize, which result from reduced expression of the *SBEIIb* gene. Analysis of amylopectin chain length distribution indicated that the SBEIIa and SBEIIb isoforms play distinct roles in determining the fine structure of amylopectin. A significant reduction in the frequency of branches in amylopectin occurred only when both SBEIIa and SBEIIb were reduced, whereas there was a significant increase in the branching frequency of amylose when SBEIIb alone was reduced. Functional interactions between SBE isoforms and a possible inhibitory role of SBEIIb on other SBE isoforms have been suggested.

The *ae* phenotype was obtained in rice by Butardo et al. (2011) by down-regulating the expression of SBEIIb using RNA silencing. For the first time, artificial microRNA (amiRNA) was used to silence RNA in rice endosperm, resulting in a more extreme starch phenotype (with distinct differences in chemistry, crystallinity, and digestibility) than when hairpin-RNA (hp-RNA) was used for suppression. However, in order to obtain very high amylose rice grains, the expression of multiple starch synthesising enzymes may need to be down-regulated simultaneously.

Zhu et al. (2012) have inhibited the expression of SBEI and SBEIIb in the *indica* rice cultivar Te-qing using a double antisense construct. One of the transgenic lines showed a large increase in amylose content, to 64.8% compared to 27.2% in the normal cultivar. Scanning electron microscopy also showed a drastic change in the morphology of the starch granules in the transgenic line. Granules of smaller size, elongated shapes and filamentous structures with large voluminous bodies with spherical or ellipsoidal profiles were present in the transgenic line, compared with polygonal shapes characteristic of the starch granules present in the normal cultivar.

Recently, Carciofi et al. (2012) have silenced all three genes encoding starch branching enzymes (*SBEI, SBEIIa and SBEIIb*) in barley by RNAi, producing transgenic lines with starch granules containing only amylose. This was achieved using a chimaeric RNAi hairpin construct with three elements targeting simultaneously each of the three SBE genes. The starch granules were irregularly shaped and showed modified thermal properties and crystallinity.

## 5 Improving cereal cell wall polysaccharides for health benefits

Increases in the contents of soluble and total DF (AX and β-glucan) are clear targets for improvement, with the latter contribution to viscosity. More subtle differences in the benefits of AX and β-glucan may also exist, but have not yet been explored. The identification, and generation, of variation in the structure and properties of these two polysaccharides will therefore allow the relationships between their structures and properties (such as viscosity and production of SCFA) that affect health benefits to be defined and exploited to produce new types of cereals with enhanced benefits.

### 5.1 Cell walls of grain tissues

AX and β-glucan account for about 70% and 20%, respectively, of the total cell wall polysaccharides in the starchy endosperm of wheat, with smaller amounts of cellulose ((1 → 4)-β-d-glucan) (about 2%) and glucomannan (about 7%) (Mares and Stone, 1973). Callose ((1 → 3)-β-d-glucan) is deposited in the early stages of endosperm development (Philippe et al., 2006; Pellny et al., 2012), and it is also possible to detect xyloglucan in the cell walls of the young developing endosperm using a specific antibody (Pellny et al., 2012). The inability to detect callose, xyloglucan or other minor cell wall polysaccharides by immunolabelling in the mature endosperm could be due to masking by the later deposition of major polysaccharides rather than their absence.

Whereas the starchy endosperm of rye is also rich in AX, the starchy endosperm cells of both barley and oats have higher contents of β-glucan than of AX. In barley, the proportions of AX, β-glucan, cellulose and glucomannan have been reported as about 20%, 70%, 3% and 2%, respectively (Fincher, 1975).

The bran fraction produced on milling comprises the pericarp and testa (often called the outer layers), the aleurone layer and, in most milling processes, also the embryo (germ). In wheat the aleurone accounts for about 6.5% of the whole grain, the outer layers for about 7–8% and the germ for about 3% (Barron et al., 2007).

The aleurone layer is the outer part of the starchy endosperm, which consists of a single layer of cells in wheat, rye and oats but usually three layers of cells in barley (except for the region adjacent to the germ which is a single layer). The aleurone cells have thick walls which account for 35–40% of the dry weight of the tissue in wheat (Barron et al., 2007). The aleurone cells of wheat, barley, rye and oats are all rich in AX, accounting for about 65% and 67% of the total in wheat and barley, respectively (Bacic and Stone, 1981). The aleurone cell walls of these two species also contain 26% and 29% β-glucan, respectively, and about 2% each of cellulose and glucomannan (Bacic and Stone, 1981).

The outer layers of the grain have been studied in most detail in wheat, where they comprise about 45–50% cell wall material (Barron et al., 2007). The major tissue is the pericarp which is similar in cell wall composition to vegetative tissues such as straw, containing about 30% cellulose, 60% AX and 12% lignin and lacking β-glucan (Stone and Morell, 2009).

### 5.2 Variation in AX structure between tissues

The AX in the cell walls of starchy endosperm cells of wheat has a low level of ferulylation, accounting for about 0.3% of the total AX, and only traces of ferulic acid dimers and no triferulate or *p*-coumaric acid (Barron et al., 2007; Saulnier et al., 2012). By contrast, the aleurone cell walls contain about 3.2% ferulate and 0.45% diferulate (both % total AX) (Saulnier et al., 2012) and *p*-coumaric acid (0.2–0.3 mg/g tissue) (Barron et al., 2007), with the latter being proposed as a biochemical marker for the tissue (Hemery et al., 2009). The AX in the outer layers varies in structure between the different layers but is generally more highly substituted than in the endosperm tissues, containing galactose and glucuronic acid, and is therefore often termed glucuronoarabinoxylan. It is also acetylated and highly ferulylated, with triferulic acid being abundant in the outer pericarp (over 1 mg/dry weight of tissue) and therefore providing a good marker for the tissue (Hemery et al., 2009).

Both AX and β-glucan are frequently fractionated into water-extractable (i.e. soluble) and water-unextractable (insoluble) forms, which are referred to as WE and WU with the total being TOT. Variation in the proportions of these fractions is discussed below.

### 5.3 Genetic variation in AX and β-glucan content and composition

**Fig. 3:**
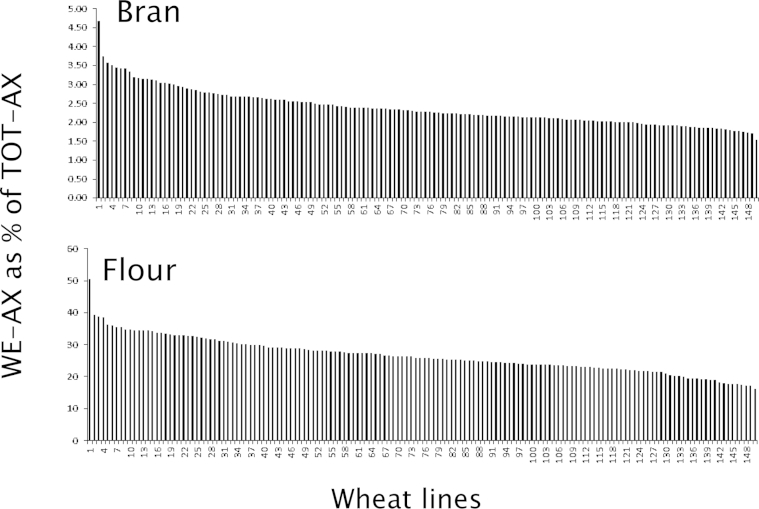
The content of WE-AX in four and bran of 150 wheat lines, expressed as % TOT-AX. Based on data reported by Gebruers et al. (2008).

A number of comparative studies have been reported of variation in the contents of AX and β-glucan in wholemeal, flour and bran of wheat (reviewed by Shewry, 2013). The most extensive of these was the EU FP6 HEALTHGRAIN study, in which 151 wheat lines (selected to represent a range of geographical origins and release dates) were grown on a single site in Hungary (Gebruers et al., 2008; Ward et al., 2008). The grain was milled to separate white flour and bran fractions. TOT-AX in bran varied from about 13 to 22% dry weight, with WE-AX varying from 0.3% to 0.85% (i.e. only 2%–5% of the TOT-AX was water-soluble, see Fig. 3). The content of TOT-AX in flour varied from 1.35% to 2.75% and WE-AX from 0.30% to 1.4%, accounting for 20%–50% of the total (Fig. 3). Hence, wheat bran is rich in insoluble AX while up to 50% of the AX in the flour is water-soluble. In the same study, wholemeal flours from the same lines contained from 0.5% to 0.95% dry weight of β-glucan.

A similar large scale study was reported by Pritchard et al. (2011), who analysed wholemeal flour of 338 hexaploid wheat lines from the Australian Winter Cereals Collection. The total content of non-starch polysaccharides ranged from 3.18% to 9.14%, TOT-AX from 2.37% to 6.58% and β-glucan from 2.0% to 14.3%. Although most of the lines had been grown at Temora (NSW) this was at different times and it is possible that some of the variation was due to the environment in the different years.

Genetic variation in the fine structure of AX in wheat flour, and in particular the proportions of monosubstituted and disubstituted xylose residues, is also revealed by endoxylanase digestion to release oligosaccharides which are then separated by HP-AEC to give an “enzyme fingerprint” (Shewry et al., 2010a; Toole et al., 2011).

Henry (1986) showed that β-glucan varied from 3.44% to 5.68% dry weight and pentosans (i.e. AX) from 4.38% to 7.79% dry weight in 17 cultivars of barley grown at three locations in Australia. The ratio of pentosans to β-glucan varied from 2.26 to 1.05, and their combined amounts from 8.5% to 11.8% dry weight. Similarly, Henry (1985) showed that total β-glucan varied from 4.03% to 5.26% in 13 cultivars and 12 lines grown on two sites in Australia over two years.

The solubility of β-glucan varies significantly between species, being about 10–15% of the total in white flour of wheat (Nemeth et al., 2010) but much higher in barley and oats.

To the best of our knowledge there is no information on whether the amounts, proportions and structures of AX and β-glucan vary significantly among aleurone tissues of different genotypes of cereals.

Andersson et al. (2013) carried out a more extensive analysis of DF components in whole grains of 129 winter wheat lines in the HEALTHGRAIN collection. This showed that total DF (analysed by the Uppsala method) varied from 11.5% to 15.5%, cellulose from 1.67% to 3.05% and fructans from 0.84% to 1.85% dry weight.

It should be noted that the HEALTHGRAIN study is currently the only large scale systematic comparison of DF in flour and bran in multiple wheat lines. Furthermore, although 151 lines were studied these only represent a fraction of the variation present in wheat; Feldman (1995) estimating the existence of at least 25,000 different wheat varieties and lines. Hence, wider germplasm screens will undoubtedly identify further variation that could be exploited.

### 5.4 Heritability and genetic control of AX and β-glucan

The studies discussed above have shown that the contents of AX and β-glucan vary widely between genotypes of wheat and other cereals in whole grain, white flour and bran fractions. However, this could result from effects of the environment as well as genetic differences, and to specific interactions between the genotype and environment. It is therefore important to determine the extent to which the differences observed are heritable. Analyses of genotypes grown on multiple sites allow the variation to be partitioned into the effects of genotype, environment and genotype × environment, with the ratio of genetic variance to total variance providing an estimate of the “broad sense heritability” of the trait. Analyses of 26 lines from the HEALTHGRAIN collection grown in six environments (sites and/or years) provided ratios of 0.39 and 0.71 for TOT-AX in bran and flour, respectively, and 0.47 and 0.59 for WE-AX in bran and flour (Gebruers et al., 2010; Shewry et al., 2010a). The ratio for total β-glucan in wholemeal was 0.51. A similarly high heritability (ratio of 0.75) was reported for WE-AX in flour by Martinant et al. (1999). However, contrasting results were reported by Li et al. (2009) who carried out multisite trials with 50 wheat lines (25 spring and 25 winter) in the USA. They showed that environment had a much greater impact than genotype on WE-AX and TOT-AX in the winter lines and on WE-AX in the spring lines. Hence the relative contributions of genotype and environment may vary with the genotypes and environments which are compared.

A series of analyses of mapping populations have allowed major quantitative trait loci (QTL) for AX to be identified in wheat. Martinant et al. (1998) analysed two mapping populations to identify a major QTL for WE-AX, extract viscosity and the ratio of arabinose to xylose in AX on chromosome 1B. Quraishi et al. (2010) combined the data from these and five more crosses (Laperche et al., 2007; Charmet et al., 2009; Quraishi et al., 2009; Perretant et al., 2000) to identify three “meta-QTL” on chromosomes 1B, 3D and 6B, while Charmet et al. (2009) reported that the QTL on 6B accounted for up to 59% of the variation in WE-AX viscosity in the two populations. Association genetic analysis of the HEALTHGRAIN diversity collection of 156 wheat lines (131 winter and 20 spring bread wheats and five *Triticum spelta* lines grown in Hungary in 2005) identified seven loci involved in WE-AX viscosity, three of which co-located with the meta-QTL located on chromosomes 1B, 3D and 6B and four additional loci on chromosomes 3A, 5B, 7A and 7B (Quraishi et al., 2010). The most significant locus identified by both these studies was that on chromosome 1B and Quraishi et al. (2010) have shown that this region of the chromosome contains four genes which may contribute to the trait. This work should therefore facilitate the development of molecular markers to allow plant breeders to screen for high flour WE-AX. Less is known about the genetic control of TOT-AX in flour or of AX structure, but Saulnier and colleagues have used endoxylanase digestion followed by HP-AEC to show that cultivars vary in AX structure, and in particular in the proportions on monosubstituted and disubstituted xylose residues (Shewry et al., 2010a, 2010b; Toole et al., 2011).

The heritability of the amount of ferulic acid bound to AX has not been determined in wheat flour, but the heritability of total bound phenolic acids (which comprised an average of 74% of total phenolic acids in these samples (Fernandez-Orozco et al., 2010)) in wholemeal of wheat is low (a ratio of 0.26) (Shewry et al., 2010a).

### 5.5 Manipulation of AX and β-glucan

The existence of genetic variation in AX amount, solubility and composition provides opportunities for plant breeders to produce improved lines using marker assisted selection. However, it is also possible to generate more precise changes in amount and composition using transgenesis and mutagenesis.

It is now well-established that the biosynthesis of β-glucan is mediated by the Cellulose synthase-like (Csl) F6 gene in barley (Burton et al., 2006) and wheat (Nemeth et al., 2010) and the over-expression of this gene under an endosperm-specific promoter in transgenic barley resulted in an increase in the β-glucan content of the grain by 80% (Burton et al., 2011). Burton et al. (2011) also showed that over-expression of the related CslF4 gene under the control of a constitutive promoter resulted in increased β-glucan in grain. Although the same enzymes appear to catalyse the formation of both 1 → 3 and 1 → 4 linkages, the expression of the two transgenes resulted in contrasting effects on the ratio of DP3 and DP4 fragments released by digestion with lichenase (indicating different distributions of the two types of linkage) (Burton et al., 2011). Consequently, transgenic expression of CslF may be used not only to increase the content of total β-glucan but also to modify its fine structure. Cseh et al. (2001) also reported the presence of increased β-glucan in a 4BS.7HL wheat-barley translocation line which contained the barley CslF6 genes in the centromeric region of chromosome 7HL, indicating that chromosome engineering could also be used to modify the content and properties of β-glucan in wheat grain.

The application of mutagenesis to manipulate grain β-glucan was reported for oats by Sikora et al. (2013). They analysed 1700 lines of a mutagenised population, obtained by EMS treatment of the variety Belinda (Chawade et al., 2010), and identified lines with altered β-glucan levels. The differences observed between the lines were great, with the β-glucan levels ranging from 1.8% to 7.5%, compared to a value of 4.9% for the parent variety. Mutants with altered β-glucan structure were also identified.

**Fig. 4:**
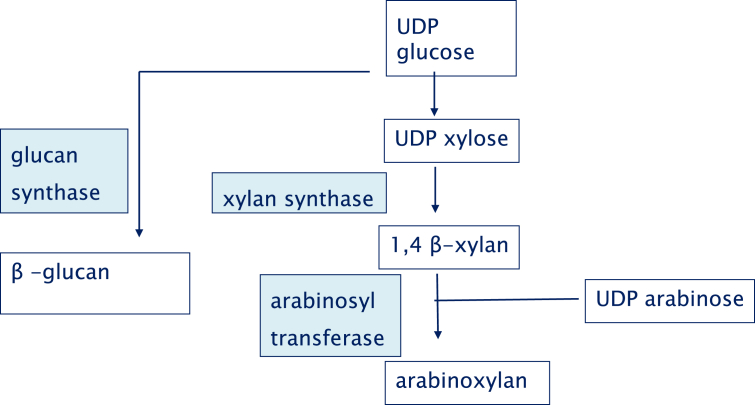
Proposed pathway of arabinoxylan synthesis. Key enzymes are in blue boxes. (For interpretation of the references to color in this figure legend, the reader is referred to the web version of this article.)

By contrast to β-glucan synthesis, the pathway of AX synthesis involves several enzymes and is still incompletely understood (Fig. 4). Mitchell et al. (2007) used bioinformatics to identify candidate genes for AX synthesis (xylan synthase and arabinosyl transferase) in the glycosyl transferase (GT) families 43, 47 and 61 and for feruloylation of arabinose in the Pfam family PF02458. These predictions have been borne out by experimental studies, in which candidate genes have been functionally characterised by expression in transgenic wheat or Arabidopsis. In wheat, most GT genes occur in multiple forms, with three homoeoalleles of each form present on the A, B and D genomes. Forms which are highly expressed in the developing starchy endosperm were therefore identified using EST databases (Mitchell et al., 2007), Affymetrix arrays (Wan et al., 2008) and RNA-Seq (Pellny et al., 2012).

Six forms of GT61 were identified as expressed in developing starchy endosperm of wheat, with GT61_1 being the most highly expressed (Pellny et al., 2012). RNAi was therefore used to suppress the expression of GT61_1 in developing starchy endosperms, using a construct designed to suppress all three homoeoalleles (Anders et al., 2012). This resulted in a specific reduction in the abundance of monosubstituted xylose residues in AX, demonstrating that the GT61_1 gene encodes an arabinosyl transfer which is specific for this position. The RNAi suppression of GT61_1 resulted in decreased TOT-AX and in reduced proportions of WE-AX in two lines (Anders et al., 2012), demonstrating that the pattern of arabinosylation can be manipulated to change AX solubility. Several other forms of GT61 are also expressed in the developing starchy endosperm (Pellny et al., 2012) and are expected to include forms encoding enzymes that catalyse the addition of arabinose to the 2 and 3 positions of disubstituted xylose residues, and ferulylated arabinose to the 3 position only.

A similar approach was used to functionally characterise the highly expressed GT43_2 and GT47_2 genes, which were putatively identified as encoding xylan synthases based on their relationship to the IRX10 and IRX9 genes, respectively, of Arabidopsis (Brown et al., 2007, 2009). In both cases RNAi suppression resulted in decreased total xylan, with no apparent effects on the proportions of monosubstituted and disubstituted xylose residues (Lovegrove et al., 2013). It is therefore probably that these two genes encode xylan synthases responsible for synthesis of the xylan backbone.

RNAi suppression of GT43_2 and GT47_2 also resulted in decreased total AX and in reduced viscosity of aqueous extracts. Determination of the concentration and intrinsic viscosity profiles of WE-AX separated by size-exclusion HPLC showed increased proportions of smaller AX chains in the transgenic lines. However, differences were also observed between GT43_2 RNAi and GT47_2 RNAi lines, with the latter having a greater effect (Lovegrove et al., 2013). The functions of other expressed forms of GT43 and GT47 (Pellny et al., 2012) are currently under investigation.

Functional characterisation of the genes responsible for ferulylation of AX has proved more elusive but transcriptomic analysis in wheat (Pellny et al., 2012) and RNAi suppression in rice (Piston et al., 2010) indicates the importance of genes from the BAHD clade of the PF02458 family.

It is clear, therefore, from these studies that the amounts and fine structures of both β-glucan and AX can be manipulated by altering the total and relative activities of biosynthetic enzymes. This could be achieved by transgenesis or mutagenesis (TILLING). However, because in wheat, most (probably all) genes encoding enzymes of cell wall biosynthesis occur in three forms, it is necessary to identify and “knock- out” at all three loci to observe effects on cell wall composition. It should also be noted that mutagenesis rarely results in increased gene expression, and is only routinely applicable where the required phenotype can result from gene knock-out(s).

## 6 Conclusions

The polysaccharides of cereal grain are not only important sources of calories in the human diet but also have health benefits related to their physical properties, digestion in the small intestine and fermentation in the colon. These contribute to the well-established health benefits associated with the regular consumption of whole grain cereal foods. There is therefore considerable interest in modifying the amounts and compositions of starch and the major non-starch polysaccharides (arabinoxylan and β-glucan) to develop new types of cereals with improved health benefits, notably reduced glycaemic index in the small intestine and improved properties as DF in the colon. This may be achieved by exploiting genetic variation in composition with the ability to generate additional variation using mutagenesis and transgenesis. The development and adoption of such varieties will allow health benefits to be delivered to large populations in low cost staple foods such as bread, noodles and pasta.

TILLING technology provides a very powerful non-GM approach to identify useful mutations which can be exploited in conventional plant breeding, as demonstrated by the increasing number of platforms developed in different crops (Sikora et al., 2011). In addition, the access to this technology is being facilitated by the establishment of public services offered by many laboratories. This technology should therefore make it possible to modify starch and NSP polysaccharide composition in a wider array of cereal crops, to generate healthier food products with increased amounts of fibre and/or resistant starch.

## References

[bib1] Akerberg A., Liljeberg H., Bjorck I. (1998). Effects of amylose/amylopectin ratio and baking conditions on resistant starch formation and glycaemic indices. J. Cereal Sci..

[bib2] Alsaffar A.A. (2011). Effect of food processing on the resistant starch content of cereals and cereal products – a review. Int. J. Food Sci. Technol..

[bib3] Anders N., Wilkinson M.D., Lovegrove A., Freeman J., Tryfona T., Pellny T.K., Weimar T., Mortimer J.C., Stott K., Baker J.M., Defoin-Platel M., Shewry P.R., Dupree P., Mitchell R.A.C. (2012). Glycosyl transferases in family 61 mediate arabinofuranosyl transfer onto xylan in grasses. Proc. Natl. Acad. Sci. U.S.A..

[bib4] Andersson A.A.M., Andersson R., Piironen V., Lampi A.-M., Nyström L., Boros D., Fras A., Gebruers K., Courtin C.M., Delcour J.A., Rakszegi M., Bedo Z., Ward J.L., Shewry P.R., Åman P. (2013). Contents of dietary fibre components and their relation to associated bioactive components in whole grain wheat samples from the HEALTHGRAIN diversity screen. Food Chem..

[bib5] Aravind N., Sissons M., Fellows C.M., Blazek J., Gilbert E.P. (2013). Optimisation of resistant starch II and III levels in durum wheat pasta to reduce *in vitro* digestibility while maintaining processing and sensory characteristics. Food Chem..

[bib6] Asp N.G. (1992). Resistant starch. Proceedings from the second plenary meeting of EURESTA: European FLAIR-Concerted Action No 11 on the physiological implications of the consumption of resistant starch in man. Eur. J. Clin. Nutr..

[bib7] Asp N.-G., Van Amelsvoort J.M.M., Hautvast J.G.A.J. (1996). Nutritional implications of resistant starch. Nutr. Res. Rev..

[bib8] Bacic A., Stone B.A. (1981). Chemistry and organization of aleurone cell wall components from wheat and barley. Aust. J. Plant Physiol..

[bib9] Barron C., Surget A., Rouau X. (2007). Relative amounts of tissues in mature wheat (*Triticum aestivum* L.) grain and their carbohydrate and phenolic acid composition. J. Cereal Sci..

[bib10] Birt D.F., Boylston T., Hendrich S., Jane J.L., Hollis J., Li L., McClelland J., Moore S., Phillips G.J., Rowling M., Schalinske K., Scott M.P., Whitley E.M. (2013). Resistant starch: promise for improving human health. Adv. Nutr..

[bib11] Blazek J., Copeland L. (2008). Pasting and swelling properties of wheat flour and starch in relation to amylose content. Carbohydr. Polym..

[bib12] Blennow A., Jensen S.L., Shaik S.S., Skryhan K., Carciofi M., Holm P.B., Hebelstrup K.H., Tanackovic V. (2013). Future cereal starch bioengineering: cereal ancestors encounter gene technology and designer enzymes. Cereal Chem..

[bib13] Blennow A., Eliasson A.C. (2004). Starch bioengineering. Starch in Food.

[bib14] Bodinham C.L., Frost G.S., Robertson M.D. (2010). Acute ingestion of resistant starch reduces food intake in healthy adults. Br. J. Nutr..

[bib15] Botticella E., Sestili F., Hernandez-Lopez A., Phillips A., Lafiandra D. (2011). High resolution melting analysis for the detection of EMS induced mutations in wheat SbeIIa genes. BMC Plant Biol..

[bib16] Boyer C.D., Preiss J. (1978). Multiple forms of starch branching enzyme of maize: evidence for independent genetic control. Biochem. Biophys. Res. Commun..

[bib17] Brown D.M., Goubet F., Wong V.W., Goodacre R., Stephens E., Dupree P., Turner S.R. (2007). Comparison of five xylan synthesis mutants reveals new insight into the mechanisms of xylan synthesis. Plant J..

[bib18] Brown D.M., Zhang Z.N., Stephens E., Dupree P., Turner S.R. (2009). Characterization of IRX10 and IRX10-like reveals an essential role in glucuronoxylan biosynthesis in Arabidopsis. Plant J..

[bib19] Brownlee I.A., Moore C., Chatfield M., Richardson D.P., Ashby P., Kuznesof S.A., Jebb S.A., Seal C.J. (2010). Markers of cardiovascular risk are not changed by increased whole-grain intake: the WHOLEheart study, a randomised, controlled dietary intervention. Br. J. Nutr..

[bib20] Burton R.A., Collins H.M., Kibble N.A.J., Smith J.A., Shirley N.J., Jobling S.A., Henderson M., Singh R.R., Pettolino F., Wilson S.M., Bird A.R., Topping D.L., Bacic A., Fincher G.B. (2011). Over-expression of specific HvCslF cellulose synthase-like genes in transgenic barley increases the levels of cell wall (1,3;1,4)-ß-d-glucans and alters their fine structure. Plant Biotechnol. J..

[bib21] Burton R.A., Wilson S.M., Hrmova M., Harvey A.J., Shirley N.J., Medhurst A., Stone B.A., Newbigin E.J., Bacic A., Fincher G.B. (2006). Cellulose synthase-like CslF genes mediate the synthesis of cell wall (1,3;1,4)-beta-d-glucans. Science.

[bib22] Butardo V.M., Fitzgerald M.A., Bird A.R., Gidley M.J., Flanagan B.M., Larroque O., Resurreccion A.P., Hunter K.C., Laidlaw H.K.C., Jobling S.A., Morell M.K., Rahman S. (2011). Impact of down-regulation of starch branching enzyme IIb in rice by artificial microRNA-and hairpin RNA-mediated RNA silencing. J. Exp. Bot..

[bib23] Butardo V.M., Daygon V.D., Colgrave M.L., Campbell P.M., Resurreccion A., Cuevas R.P., Jobling S.A., Tetlow I., Rahman S., Morell M., Fitzgerald M. (2012). Biomolecular analyses of starch and starch granule proteins in the high-amylose rice mutant goami 2. J. Agric. Food Chem..

[bib24] Campbell M.R., Jane J., Pollak L., Blanco M., O’Brien A. (2007). Registration of maize germplasm line GEMS-0067. J. Plant Registr..

[bib25] Candido E.P.M., Reeves R., Davies J.R. (1978). Sodium butyrate inhibits histone deacetylation in cultured cells. Cell.

[bib26] Carciofi M., Blennow A., Jensen S.L., Shaik S.S., Henriksen A., Buléon A., Holm P.B., Hebelstrup K.H. (2012). Concerted suppression of all starch branching enzyme genes in barley produces amylose-only starch granules. BMC Plant Biol..

[bib27] Charmet G., Masood-Quraishi U., Ravel C., Romeuf I., Balfourier F., Perretant M.R., Joseph J.L., Rakszegi M., Guillon F., Sado P.E., Bedo Z., Saulnier L. (2009). Genetics of dietary fibre in bread wheat. Euphytica.

[bib28] Chawade A., Sikora P., Brautigam M., Larsson M., Vivekanand V., Nakash M.A., Chen T., Olsson O. (2010). Development and characterization of an oat TILLING-population and identification of mutations in lignin and beta-glucan biosynthesis genes. BMC Plant Biol..

[bib29] Cho S.S., Qi L., Fahey G.C., Klurfeld D.M. (2013). Consumption of cereal fiber, mixtures of whole grains and bran, and whole grains and risk reduction in type 2 diabetes, obesity, and cardiovascular disease. Am. J. Clin. Nutr..

[bib30] Comai L., Young K., Till B.J., Reynolds S.H., Greene E.A., Codomo C.A., Enns L.C., Johnson J.E., Burtner C., Odden A.R., Henikoff S. (2004). Efficient discovery of DNA polymorphisms in natural populations by Ecotilling. Plant J..

[bib31] Copeland L., Blazek J., Salman H., Tang M.C. (2009). Form and functionality of starch. Food Hydrocoll..

[bib32] Creech R.G. (1965). Genetic control of carbohydrate synthesis in maize endosperm. Genetics.

[bib33] Creech R.G., McArdle F.J., Kramer H.H. (1963). Genetic control of carbohydrate type and quantity in maize kernels. Maize Genet. Coop. Newsl..

[bib34] Cseh A., Kruppa K., Molnar I., Rakszegi M., Dolezel J., Molnar-Lang M. (2001). Characterization of a new 4BS.7HL wheat-barley translocation line using GISH, FISH, and SSR markers and its effect on the bega-glucan content of wheat. Genome.

[bib35] Cui W., Wood P.J., Blackwell B., Nikiforuk J. (2000). Physicochemical properties and structural characterization by two-dimensional NMR spectroscopy of wheat β-d-glucan – comparison with other cereal β-d-glucans. Carbohydr. Polym..

[bib36] De Natale C., Minerva V., Patti L., Mazzarella R., Ciano O., Maione S., Luongo D., Naviglio D., Marotta G., Turco S., Ciati R., Melegari C., Rivellese A.A., Riccardi G. (2012). Effects of baked products enriched with n-3 fatty acids, folates, β-glucans, and tocopherol in patients with mild mixed hyperlipidemia. J. Am. Coll. Nutr..

[bib37] Delzenne N.M., Neyrinck A.M., Cani P.D. (2011). Modulation of the gut microbiota by nutrients with prebiotic properties: consequences for host health in the context of obesity and metabolic syndrome. Microb. Cell. Factor..

[bib38] Delzenne N.M., Neyrinck A.M., Cani P.D. (2013). Gut microbiota and metabolic disorders: how prebiotic can work?. Br. J. Nutr..

[bib39] Dunwell J.M. (2014). Transgenic cereals: current status and future prospects. J. Cereal Sci..

[bib40] Dunwell J.M., Wetten A.C. (2012). Transgenic Plants. Methods and Protocols.

[bib41] El Khoury D., Cuda C., Luhovyy B.L., Anderson G.H. (2012). Beta glucan: health benefits in obesity and metabolic syndrome. J. Nutr. Metabol..

[bib42] Englyst H.N., Cummings J.H. (1985). Digestion of the polysaccharides of some cereal foods in the human small intestine. Am. J. Clin. Nutr..

[bib43] Eslick R.F., Ullrich S.E. (1977). A characterization of three high lysine mutants in relation to their normal isotypes and their environmental response. Barley Newsl..

[bib44] Fardet A. (2010). New hypotheses for the health-protective mechanisms of whole-grain cereals: what is beyond fibre?. Nutr. Res. Rev..

[bib45] Feldman M., Smartt J., Simmonds N.W. (1995). Wheats. Evolution of Crop Plants.

[bib46] Fergason V., Hallauer A.R. (2001). High amylose and waxy corns. Speciality Corns.

[bib47] Fernandez-Orozco R., Li L., Harflett C., Shewry P.R., Ward J.L. (2010). Effects of environment and genotype on phenolic acids in wheat in the HEALTHGRAIN diversity screen. J. Agric. Food Chem..

[bib48] Fincher G. (1975). Morphology and chemical composition of barley endosperm cell walls. J. Inst. Brew..

[bib49] Fujita N., Kubo A., Suh D.S., Wong K.S., Jane J.L., Ozawa K., Takaiwa F., Inaba Y., Nakamura Y. (2003). Antisense inhibition of isoamylase alters the structure of amylopectin and the physicochemical properties of starch in rice endosperm. Plant Cell. Physiol..

[bib50] Gebruers K., Dornez E., Boros D., Frás A., Dynkowska W., Bedő Z., Rakszegi M., Delcour J.A., Courtin C.M. (2008). Variation in the content of dietary fiber and components thereof in wheats in the HEALTHGRAIN diversity screen. J. Agric. Food Chem..

[bib51] Gebruers K., Dornez E., Bedo Z., Rakszegi M., Fras A., Boros D., Courtin C.M., Delcour J.A. (2010). Environment and genotype effects on the content of dietary fiber and its components in wheat in the HEALTHGRAIN diversity screen. J. Agric. Food Chem..

[bib52] Giacco R., Della Pepa G., Luongo D., Riccardi G. (2011). Whole grain intake in relation to body weight: from epidemiological evidence to clinical trials. Nutr. Metabol. Cardiovasc. Dis..

[bib53] Giacco R., Clemente G., Cipriano D., Luongo D., Viscovo D., Patti L., Di Marino L., Giacco A., Naviglio D., Bianchi M.A., Ciati R., Brighenti F., Rivellese A.A., Riccardi G. (2010). Effects of the regular consumption of wholemeal wheat foods on cardiovascular risk factors in healthy people. Nutr. Metabol. Cardiovasc. Dis..

[bib54] Giacco R., Lappi J., Costabile G., Kolehmainen M., Schwab U., Landberg R., Uusitupa M., Poutanen K., Pacini G., Rivellese A.A., Riccardi G., Mykkanen H. (2013). Effects of rye and whole wheat versus refined cereal foods on metabolic risk factors: a randomized controlled two-centre intervention study. Clin. Nutr..

[bib55] Gil A., Ortega R.M., Maldonado J. (2011). Wholegrain cereals and bread: a duet of the Mediterranean diet for the prevention of chronic diseases. Pub. Health Nutr..

[bib56] Granfeldt Y., Drews A., Bjorck I. (1995). Arepas made from high amylose corn flour produce favorable low glucose and insulin responses in healthy humans. J. Nutr..

[bib57] Graybosch R.A. (1998). Waxy wheats: origin, properties, and prospects. Trends Food Sci. Technol..

[bib58] Hallström E., Sestili F., Lafiandra D., Björck I., Östman E. (2011). A novel wheat variety with elevated content of amylose increases resistant starch formation and may beneficially influence glycaemia in healthy subjects. Food Nutr. Res..

[bib59] Hannah L.C., James M. (2008). The complexities of starch biosynthesis in cereal endosperms. Curr. Opin. Biotechnol..

[bib60] Hasjim J., Lee S.-O., Hendrich S., Setiawan S., Ai Y., Jane J. (2010). Characterization of novel resistant-starch and its effects on postprandial plasma-glucose and insulin responses. Cereal Chem..

[bib61] Hazard B., Zhang X., Colasuonno P., Uauy C., Beckles D.M., Dubcovsky J. (2012). Induced mutations in the Starch Branching Enzyme II (SBEII) genes increase amylose and resistant starch content in durum wheat. Crop Sci..

[bib62] Hedman K.D., Boyer C.D. (1982). Gene dosage at the amylose-extender locus of maize: effects on the levels of starch branching enzymes. Biochem. Genet..

[bib63] Hemery Y., Lullien-Pellerin V., Rouau X., Abecassis J., Samson M.-F., Åman P., von Reding W., Spoerndli C., Barron C. (2009). Biochemical markers: efficient tools for the assessment of wheat grain tissue proportions in milling fractions. J. Cereal Sci..

[bib64] Henry R.J. (1985). A comparative study of the total β-glucan contents of some Australian barleys. Aust. J. Exp. Agric..

[bib65] Henry R.J. (1986). Genetic and environmental variation in the pentosan and β-glucan contents of barley, and their relation to malting quality. J. Cereal Sci..

[bib66] Hogg A.C., Gause K., Hofer P., Martin J.M., Graybosch R.A., Hansen L.E., Giroux M.J. (2013). Creation of a high-amylose durum wheat through mutagenesis of starch synthase II (SSIIa). J. Cereal Sci..

[bib67] Jiang H., Jane J.-L., Acevedo D., Green A., Shinn G., Schrenker D., Srichuwong S., Campbell M., Wu Y. (2010). Variations in starch physicochemical properties from a generation-means analysis study using Amylomaize V and VII parents. J. Agric. Food Chem..

[bib68] Jeon J.-S., Ryoo N., Hahn T.-R., Walia H., Nakamura Y. (2010). Starch biosynthesis in cereal endosperm. Plant Physiol. Biochem..

[bib69] Jones H.D., Shewry P.R. (2008). Transgenic Wheat, Barley and Oats: Production and Characterisation.

[bib70] Jones H.D., Sparks C.A., Shewry P.R., Khan K., Shewry P.R. (2009). Wheat: Chemistry and Technology.

[bib71] Juvonen K.R., Salmenkallio-Marttila M., Lyly M., Liukkonen K.H., Lähteenmäki L., Laaksonen D.E., Uusitupa M.I., Herzig K.H., Poutanen K.S., Karhunen L.J. (2011). Semisolid meal enriched in oat bran decreases plasma glucose and insulin levels, but does not change gastrointestinal peptide responses or short-term appetite in healthy subjects. Nutr. Metabol. Cardiovasc. Dis..

[bib72] Kadaru S.B., Yadav A.S., Fjellstrom R.G., Oard J.H. (2006). Alternative ecotilling protocol for rapid, cost-effective single-nucleotide polymorphism discovery and genotyping in rice (*Oryza sativa* L.). Plant Mol. Biol. Report..

[bib73] Kang H.J., Hwang I.K., Kim K.S., Choi H.C. (2003). Comparative structure and physicochemical properties of IIpumbyeo, a high quality japonica rice, and its mutant, Suweon 464. J. Agric. Food Chem..

[bib74] Keeling P.L., Myers A.M. (2010). Biochemistry and genetics of starch synthesis. Annu. Rev. Food Sci. Technol..

[bib75] Keenan M.J., Martin R.J., Raggio A.M., McCutcheon K.L., Brown I.L., Birkett A., Newman S.S., Skaf J., Hegsted M., Tulley R.T., Blair E., Zhou J. (2012). High-amylose resistant starch increases hormones and improves structure and function of the gastrointestinal tract: a microarray study. J. Nutrigenet. Nutrigenom..

[bib76] Keenan M.J., Zhou J., McCutcheon K.L., Raggio A.M., Bateman H.G., Todd E., Jones C.K., Tulley R.T., Melton S., Martin R.J., Hegsted M. (2006). Effects of resistant starch, a non-digestible fermentable fiber, on reducing body fat. Obesity.

[bib77] Konik-Rose C., Thistleton J., Chanvrier H., Tan I., Halley P., Gidley M., Kosar-Hashemi B., Wang H., Larroque O., Ikea J., McMaugh S., Regina A., Rahman S., Morell M., Li Z. (2007). Effects of starch synthase IIa gene dosage on grain, protein and starch in endosperm of wheat. Theor. Appl. Genet..

[bib78] Kubo A., Fujita N., Harada K., Matsuda T., Satoh H., Nakamura Y. (1999). The starch debranching enzymes isoamylase and pullulanase are both involved in amylopectin biosynthesis in rice endosperm. Plant Physiol..

[bib79] Lafiandra D., Sestili F., D'Ovidio R., Janni M., Botticella E., Ferrazzano G., Silvestri M., Ranieri R., DeAmbrogio E. (2010). Approaches for modification of starch composition in durum wheat. Cereal Chem..

[bib80] Laperche A., Brancourt-Hulmel M., Heumez E., Gardet O., Hanocq E., Devienne-Barret F., Le Gouis J. (2007). Using genotype x nitrogen interaction variables to evaluate the QTL involved in wheat tolerance to nitrogen constraints. Theor. Appl. Genet..

[bib81] Lazaridou A., Biliaderis C.G. (2007). Molecular aspects of cereal β-glucan functionality: physical properties, technological applications and physiological effects. J. Cereal Sci..

[bib82] Lazaridou A., Biliaderis C.G., Micha-Screttas M., Steele B.R. (2004). A comparative study on structure-function relations of mixed linkage (1-3), (1-4) linear b-d-glucans. Food Hydrocoll..

[bib83] Le Leu R.K., Brown I.L., Hu Y., Bird A.R., Jackson M., Esterman A., Young G.P. (2005). A symbiotic combination of resistant starch and *Bifidobacterium lactis* facilitates apoptotic deletion of carcinogen damaged cells in rat colon. J. Nutr..

[bib84] Li Z., Chu X., Mouille G., Yan L., Kosar-Hashemi B., Hey S., Napier J., Shewry P., Clarke B., Appels R., Morell M.K., Rahman S. (1999). The localization and expression of the class II starch synthases of wheat. Plant Physiol..

[bib85] Li L., Jiang H., Campbell M., Blanco M., Jane J. (2008). Characterization of maize amylose-extender (*ae*) mutant starches: Part I. Relationship between resistant starch contents and molecular structures. Carbohydr. Polym..

[bib86] Li S., Morris C.F., Bettge A.D. (2009). Genotype and environment variation for arabinoxylans in hard winter and spring wheats of the US Pacific Northwest. Cereal Chem..

[bib87] Li Z., Li D., Du X., Wang H., Larroque O., Jenkins C.L.D., Jobling S.A., Morell M.K. (2011). The barley amo1 locus is tightly linked to the starch synthase IIIa gene and negatively regulates expression of granule-bound starch synthetic genes. J. Exp. Bot..

[bib88] Li W., Cui S.W., Kakuda Y. (2006). Extraction, fractionation, structural and physical characterization of wheat β-d-glucans. Carbohydr. Polym..

[bib89] Liljeberg H.G., Björck I.M. (1996). Delayed gastric emptying rate as a potential mechanism for lowered glycemia after eating sourdough bread: studies in humans and rats using test products with added organic acids or an organic salt. Am. J. Clin. Nutr..

[bib90] Liu F., Ahmed Z., Lee E.A., Donner E., Liu Q., Ahmed R., Morell M.K., Emes M.J., Tetlow I.J. (2012). Allelic variants of the amylose extender mutation of maize demonstrate phenotypic variation in starch structure resulting from modified protein–protein interactions. J. Exp. Bot..

[bib91] Lovegrove A., Wilkinson M.D., Freeman F., Pellny T.K., Tosi P., Saulnier L., Shewry P.R., Mitchell R.A.C. (2013). RNA interference suppression of genes in glycosyl transferase families 43 and 47 in wheat starchy endosperm causes large decreases in arabinoxylan content. Plant Physiol..

[bib92] Mann J., Cummings J.H., Englyst H.N., Key T., Liu S., Riccardi G., Summerbell C., Uauy R., van Dam R.M., Venn B., Vorster H.H., Wiseman M. (2007). FAO/WHO scientific update on carbohydrates in human nutrition: conclusions. Eur. J. Clin. Nutr..

[bib93] Mares D.J., Stone B.A. (1973). Studies on wheat endosperm. I. Chemical composition and ultrastructure of the cell walls. Aust. J. Biol. Sci..

[bib94] Martinant J.P., Billot A., Bouguennec A., Charmet G., Saulnier L., Branlard G. (1999). Genetic and environmental variations in water-extractable arabinoxylans content and flour extract viscosity. J. Cereal Sci..

[bib95] Martinant J.P., Cadelen T., Billot A., Chartier S. (1998). Genetic analysis of water-extractable arabinoxylans in bread wheat endosperm. Theor. Appl. Genet..

[bib96] McCallum C.M., Comai L., Greene E.A., Henikoff S. (2000). Targeting induced local lesions in genomes (TILLING) for plant functional genomics. Plant Physiol..

[bib97] Merritt N.R. (1967). A new strain of barley with starch of high amylase content. J. Inst. Brew..

[bib98] Miura H., Tanii S., Nakamura T., Watanabe N. (1994). Genetic control of amylose content in wheat endosperm starch and differential effects of three *Wx* genes. Theor. Appl. Genet..

[bib99] Mizuno K., Kawasaki T., Shimada H., Satoh H., Kobayashi E., Okumura S., Arai Y., Baba T. (1993). Alteration of the structural properties of starch components by the lack of an isoform of starch branching enzyme in rice. J. Biol. Chem..

[bib100] Morell M.K., Kosar-Hashemi B., Cmiel M., Samuel M.S., Chandler P., Rahman S., Buleon A., Batey I.L., Li Z. (2003). Barley sex6 mutants lack starch synthase lla activity and contain a starch with novel properties. Plant J..

[bib101] Morell M.K., Konik-Rose C., Ahmed R., Li Z., Rahman S. (2004). Synthesis of resistant starches in plants. J. AOAC Int..

[bib102] Mitchell R.A.C., Dupree P., Shewry P.R. (2007). A novel bioinformatics approach identifies candidate genes for the synthesis and feruloylation of arabinoxylan. Plant Physiol..

[bib103] NDNS (2011). National Diet and Nutrition Survey.

[bib104] Nemeth C., Freeman J., Jones H.D., Sparks C., Pellny T.K., Wilkinson M.D., Dunwell J., Andersson A.A.M., Aman P., Guillon F., Saulnier L., Mitchell R.A.C., Shewry P.R. (2010). Down-regulation of the CSLF6 gene results in decreased (1,3;1,4)-beta-d-glucan in endosperm of wheat. Plant Physiol..

[bib105] Nugent A.P. (2005). Health properties of resistant starch. Nutr. Bull..

[bib106] Parillo M., Riccardi G. (2004). Diet composition and the risk of type 2 diabetes epidemiological and clinical evidence. Br. J. Nutr..

[bib107] Pellny T.K., Lovegrove A., Freeman J., Tosi P., Love C.G., Knox J.P., Shewry P.R., Mitchell R.A.C. (2012). Cell walls of developing wheat starchy endosperm: comparison of composition and RNA-Seq transcriptome. Plant Physiol..

[bib108] Pereira M.A., Ludwig D.S. (2001). Dietary fiber and body-weight regulation. Observations and mechanisms. Pediatric Clin. N. Am..

[bib109] Perretant M.R., Cadalen T., Charmet G., Sourdille P., Nicolas P., Boeuf C., Tixier M.H., Branlard G., Bernard S., Bernard M. (2000). QTL analysis of bread-making quality in wheat using a doubled haploid population. Theor. Appl. Genet..

[bib110] Philippe S., Saulnier L., Guillon F. (2006). Arabinoxylan and (1 → 3),(1 → 4)-beta-glucan deposition in cell walls during wheat endosperm development. Planta.

[bib111] Piston F., Uauy C., Fu L.H., Langston J., Labavitch J., Dubcovsky J. (2010). Down-regulation of four putative arabinoxylan feruloyl transferase genes from family PF02458 reduces ester-linked ferulate content in rice cell walls. Planta.

[bib112] Poli A., Marangoni F., Paoletti R., Mannarino E., Lupattelli G., Notarbartolo A., Aureli P., Bernini F., Cicero A., Gaddi A., Catapano A., Cricelli C., Gattone M., Marrocco W., Porrini M., Stella R., Vanotti A., Volpe M., Volpe R., Cannella C., Pinto A., Del Toma E., La Vecchia C., Tavani A., Manzato E., Riccardi G., Sirtori C., Zambon A. (2008). Non-pharmacological control of plasma cholesterol levels. Nutr. Metabol. Cardiovasc. Dis..

[bib113] Pritchard J.R., Lawrence G.J., Larroque O., Li Z., Laidlaw H.K.C., Morell M.K., Rahman S. (2011). A survey of β-glucan and arabinoxylan content in wheat. J. Sci. Food Agric..

[bib114] Quraishi U.-M., Abrouk M., Bolot S., Pont C., Throude M., Guilhot N., Confolent C., Bortolini F., Praud S., Murigneux A., Charmet G., Salse J. (2009). Genomics in cereals: from genome-wide conserved orthologous set (COS) sequences to candidate genes for trait dissection. Funct. Integr. Genom..

[bib115] Quraishi U.-M., Murat F., Abrouk M., Pont C., Confolent C., Oury F.X., Ward J., Boros D., Gebruers K., Delcour J.A., Courtin C.M., Bedo Z., Saulnier L., Guillon F., Balzergue S., Shewry P.R., Feuillet C., Charmet G., Salse J. (2010). Combined meta-genomics analyses unravel candidate genes for the grain dietary fibre content in bread wheat (*Triticum aestivum* L.). Funct. Integr. Genom..

[bib116] Rahman S., Bird A., Regina A., Li Z., Philippe Ral J., McMaugh S., Topping D., Morell M. (2007). Resistant starch in cereals: exploiting genetic engineering and genetic variation. J. Cereal Sci..

[bib117] Regina A., Bird A., Topping D., Bowden S., Freeman J., Barsby T., Kosar-Hashemi B., Li Z., Rahman S., Morell M. (2006). High-amylose wheat generated by RNA interference improves indices of large-bowel health in rats. Proc. Natl. Acad. Sci. U.S.A..

[bib118] Regina A., Kosar-Hashemi B., Ling S., Li Z.Y., Rahman S., Morell M. (2010). Control of starch branching in barley defined through differential RNAi suppression of starch branching enzyme IIa and IIb. J. Exp. Bot..

[bib119] Riccardi G., Aggett P., Brighenti F., Delzenne N., Frayn K., Nieuwenhuizen A., Pannemans D., Theis S., Tuijtelaars S., Vessby B. (2004). PASSCLAIM–body weight regulation, insulin sensitivity and diabetes risk. Eur. J. Nutr..

[bib120] Riccardi G., Capaldo B., Vaccaro O. (2005). Functional foods in the management of obesity and type 2 diabetes. Curr. Opin. Clin. Nutr. Metabol. Care.

[bib121] Riccardi G., Rivellese A.A., Giacco R. (2008). Role of glycemic index and glycemic load in the healthy state, in prediabetes, and in diabetes. Am. J. Clin. Nutr..

[bib122] Riccardi G., Rivellese A.A. (2000). Dietary treatment of the metabolic syndrome-the optimal diet. Br. J. Nutr..

[bib123] Roberfroid M., Gibson G.R., Hoyles L., McCartney A.L., Rastall R., Rowland I., Wolvers D., Watzl B., Szajewska H., Stahl B., Guarner F., Respondek F., Whelan K., Coxam V., Davicco M.J., Léotoing L., Wittrant Y., Delzenne N.M., Cani P.D., Neyrinck A.M., Meheust A. (2010). Prebiotic effects: metabolic and health benefits. Br. J. Nutr..

[bib124] Rosén L.A., Östman E.M., Björck I.M. (2011). Postprandial glycemia, insulinemia, and satiety responses in healthy subjects after whole grain rye bread made from different rye varieties. J. Agric. Food Chem..

[bib125] Sajilata M.G., Singhal R.S. (2005). Specialty starches for snack foods. Carbohydr. Polym..

[bib126] Sajilata M.G., Singhal R.S., Kulkarni P.R. (2006). Resistant starch: a review. Compr. Rev. Food Sci. Food Saf..

[bib127] Sano Y. (1984). Differential regulation of waxy gene expression in rice endosperm. Theor. Appl. Genet..

[bib128] Saris W.H., Asp N.G., Björck I., Blaak E., Bornet F., Brouns F., Frayn K.N., Fürst P., Riccardi G., Roberfroid M., Vogel M. (1998). Functional food science and substrate metabolism. Br. J. Nutr..

[bib129] Saulnier L., Guillon F., Chateigner-Boutin A.-L. (2012). Cell wall deposition and metabolism in wheat grain. J. Cereal Sci..

[bib130] Sestili F., Botticella E., Bedo Z., Phillips A., Lafiandra D. (2010). Production of novel allelic variation for genes involved in starch biosynthesis through mutagenesis. Mol. Breed..

[bib131] Sestili F., Janni M., Doherty A., Botticella E., D'Ovidio R., Masci S., Jones H.D., Lafiandra D. (2010). Increasing the amylose content of durum wheat through silencing of the *SBEIIa* genes. BMC Plant Biol..

[bib132] Sharma A., Yadav B.S., Ritika (2008). Resistant starch: physiological roles and food applications. Food Rev. Int..

[bib133] Shewry P.R., Piironen V., Lampi A.-M., Edelmann M., Kariluoto S., Nurmi T., Fernandez-Orozco R., Ravel C., Charmet G., Andersson A.A.M., Åman P., Boros D., Gebruers K., Dornez E., Courtin C.M., Delcour J.A., Rakszegi M., Bedo Z., Ward J.L. (2010). The HEALTHGRAIN wheat diversity screen: effects of genotype and environment on phytochemicals and dietary fiber components. J. Agric. Food Chem..

[bib134] Shewry P.R., Saulnier L., Guillon F., Gebruers K., Courtin C., Delcour J., Toole G., Boros D., Salse J., Ravel C., Mills E.N.C., Ward J.L., Charmet G., van der Kamp J.W., Jones J., McCleary B., Topping D. (2010). Improving the benefits of wheat as a source of dietary fibre. Dietary Fibre: New Frontiers for Food and Health.

[bib135] Shewry P.R., Delcour J.A., Poutanen K. (2013). Improving the content and composition of dietary fibre in wheat. Fibre-rich and Wholegrain Foods.

[bib136] Sikora P., Chawade A., Larsson M., Olsson J., Olsson O. (2011). Mutagenesis as a tool in plant genetics, functional genomics, and breeding. Int. J. Plant Genom..

[bib137] Sikora P., Tosh S.M., Brummer Y., Olsson O. (2013). Identification of high β-glucan oat lines and localization and chemical characterization of their seed kernel β-glucans. Food Chem..

[bib138] Slade A.J., Fuerstenberg S.I., Loeffler D., Steine M.N., Facciotti D. (2005). A reverse genetic, nontransgenic approach to wheat crop improvement by TILLING. Nat. Biotechnol..

[bib139] Slade A.J., McGuire C., Loeffler D., Mullenberg J., Skinner W., Fazio G., Holm A., Brandt K.M., Stein M.N., Goodstal J.F., Knauf V.C. (2012). Development of high amylose wheat through TILLING. BMC Plant Biol..

[bib140] Slavin J., Green H. (2007). Dietary fibre and satiety. Nutr. Bull..

[bib141] Slavin J. (2013). Fiber and prebiotics: mechanisms and health benefits. Nutrients.

[bib142] Soh N.H., Sissons M.J., Turner M.A. (2006). Effect of starch granule size distribution and elevated amylose content on durum dough rheology and spaghetti cooking quality. Cereal Chem..

[bib143] Stahl Y., Coates S., Bryce J.H., Morris P.C. (2004). Antisense downregulation of the barley limit dextrinase inhibitor modulates starch granule size distribution, starch composition and amylopectin structure. Plant J..

[bib144] Steer T., Thane C., Stephen A., Jebb S. (2008). Bread in the diet: consumption and contribution to nutrient intakes of British adults. Proc. Nutr. Soc..

[bib145] Stone B., Morell M.K., Khan K., Shewry P.R. (2009). Carbohydrates. Wheat: Chemistry and Technology.

[bib146] Tester R.F., Karkalas J., Qi X. (2004). Starch – composition, fine structure and architecture. J. Cereal Sci..

[bib147] Tester R.F., Qi X., Karkalas J. (2006). Hydrolysis of native starches with amylases. Animal Feed Sci. Technol..

[bib148] Tetlow I.J. (2011). Starch biosynthesis in developing seeds. Seed Sci. Res..

[bib149] Themeier H., Hollmann J., Neese U., Lindhauer M.G. (2005). Structural and morphological factors influencing the quantification of resistant starch II in starches of different botanical origin. Carbohydr. Polym..

[bib150] Thompson D.B. (2000). Strategies for the manufacture of resistant starch. Trends Food Sci. Technol..

[bib151] Tian Z., Qian Q., Liu Q., Yan M., Liu X., Yan C., Liu G., Gao Z., Tang S., Zeng B., Wang Y., Yu Y., Gu M., Li J. (2009). Allelic diversities in rice starch biosynthesis lead to a diverse array of rice eating and cooking qualities. Proc. Natl. Acad. Sci. U.S.A..

[bib152] Toden S., Bird A.R., Topping D.L., Conlon M.A. (2007). Dose-dependent reduction of dietary protein induced colonocyte DNA damage by resistant starch in rats correlates more highly with caecal butyrate than with other short chain fatty acids. Cancer Biol. Ther..

[bib153] Toole G.A., Le Gall G., Colquhoun I.J., Johnson P., Bedő Z., Saulnier L., Shewry P.R., Mills E.N.C. (2011). Spectroscopic analysis of diversity of arabinoxylan structures in endosperm cell walls of wheat varieties (*Triticum aestivum*) in the HEALTHGRAIN diversity collection. J. Agric. Food Chem..

[bib154] Topping D. (2007). Cereal complex carbohydrates and their contribution to human health. J. Cereal Sci..

[bib155] Topping D.L., Clifton P.M. (2001). Short-chain fatty acids and human colonic function: roles of resistant starch and nonstarch polysaccharides. Physiol. Rev..

[bib156] Topping D.L., Bajka B.H., Bird A.R., Clarke J.M., Cobiac L., Conlon M.A., Morell M.K., Toden S. (2008). Resistant starches as a vehicle for delivering health benefits to the human large bowel. Microb. Ecol. Health Dis..

[bib157] Uauy C., Paraiso F., Colasuonno P., Tran R.K., Tsai H., Berardi S., Comai L., Dubcovsky J. (2009). A modified TILLING approach to detect induced mutations in tetraploid and hexaploid wheats. BMC Plant Biol..

[bib158] Umemoto T., Yano M., Satoh H., Shomura A., Nakamura Y. (2002). Mapping of a gene responsible for the difference in amylopectin structure between japonica-type and indica-type rice varieties. Theor. Appl. Genet..

[bib159] Vineyard M.L., Bear R.P. (1952). Amylose content. Maize Genet. Coop. Newsl..

[bib160] Vineyard M.L., Bear R.P., MacMasters M.M., Deatherage W.L. (1958). Development of “amylomaize”-corn hybrids with high amylose starch: I. Genetic considerations. Agron. J..

[bib161] Wan Y.F., Poole R.L., Huttly A.K., Toscano-Underwood C., Feeney K., Welham S., Gooding M.J., Mills C., Edwards K.J., Shewry P.R., Mitchell R.A.C. (2008). Transcriptome analysis of grain development in hexaploid wheat. BMC Genom..

[bib162] Ward J.L., Poutanen K., Gebruers K., Pironen V., Lampi A.M., Nyström L., Andersson A.A.M., Åman P., Boros D., Rakszegi M., Bedő Z., Shewry P.R. (2008). The HEALTHGRAIN cereal diversity screen: concept, results, and prospects. J. Agric. Food Chem..

[bib163] Waring S. (2005). Functionality of resistant starch in food applications. http://www.foodinnovations.com/pdf/functresist.pdf.

[bib164] Wirstrom T., Hilding A., Gu H.F., Ostenson C.G., Bjorklund A. (2013). Consumption of whole grain reduces risk of deteriorating glucose tolerance, including progression to prediabetes. Am. J. Clin. Nutr..

[bib165] Yamamori M. (2009). Amylose content and starch properties generated by five variant *Wx* alleles for granule-bound starch synthase in common wheat (*Triticum aestivum* L.). Euphytica.

[bib166] Yamamori M., Endo T.R. (1996). Variation of starch granule proteins and chromosome mapping of their coding genes in common wheat. Theor. Appl. Genet..

[bib167] Yamamori M., Fujita S., Hayakawa K., Matsuki J., Yasui T. (2000). Genetic elimination of a starch granule protein, SGP-1, of wheat generates an altered starch with apparent high amylose. Theor. Appl. Genet..

[bib168] Yano M., Okuno K., Kawakami J., Satoh H., Omura T. (1985). High amylose mutants of rice, *Oryza sativa L*. Theor. Appl. Genet..

[bib169] Yoo S.H., Jane J. (2002). Structural and physical characteristics of waxy and other wheat starches. Carbohydr. Polym..

[bib170] Yang C.Z., Shu X.L., Zhang L.L., Wang X.Y., Zhao H.J., Ma C.X., Wu D.X. (2006). Starch properties of mutant rice high in resistant starch. J. Agric. Food Chem..

[bib171] Zeeman S.C., Kossmann J., Smith A.M. (2010). Starch: its metabolism, evolution, and biotechnological modification in plants. Annu. Rev. Plant Biol..

[bib172] Zhu L., Gu M., Meng X., Cheung S.C., Yu H., Huang J., Sun Y., Shi Y., Liu Q. (2012). High-amylose rice improves indices of animal health in normal and diabetic rats. Plant Biotechnol. J..

